# Kinetic fingerprinting of metabotropic glutamate receptors

**DOI:** 10.1038/s42003-023-04468-z

**Published:** 2023-01-27

**Authors:** Taulant Kukaj, Christian Sattler, Thomas Zimmer, Ralf Schmauder, Klaus Benndorf

**Affiliations:** Institute of Physiology II, Jena University Hospital, Friedrich Schiller University Jena, 07743 Jena, Germany

**Keywords:** Kinetics, G protein-coupled receptors, Molecular neuroscience, Molecular biophysics, Molecular conformation

## Abstract

Dimeric metabotropic glutamate receptors (mGluRs) are abundantly expressed in neurons. In mammals, eight subunit isoforms, mGluR1-8, have been identified, forming the groups I, II, and III. We investigated receptor dimerization and kinetics of these mGluR isoforms in excised membrane patches by FRET and confocal patch-clamp fluorometry. We show that 5 out of 8 homodimeric receptors develop characteristic glutamate-induced on- and off-kinetics, as do 11 out of 28 heterodimers. Glutamate-responsive heterodimers were identified within each group, between groups I and II as well as between groups II and III, but not between groups I and III. The glutamate-responsive heterodimers showed heterogeneous activation and deactivation kinetics. Interestingly, mGluR7, not generating a kinetic response in homodimers, showed fast on-kinetics in mGluR2/7 and mGluR3/7 while off-kinetics retained the speed of mGluR2 or mGluR3 respectively. In conclusion, glutamate-induced conformational changes in heterodimers appear within each group and between groups if one group II subunit is present.

## Abstract (web summary)

FRET and confocal patch-clamp fluorometry in oocytes is used to analyze glutamateinduced conformational changes and dimerization of mGluR 1-8 homo- and heterodimers.

## Introduction

Metabotropic glutamate receptors (mGluRs) are G protein-coupled receptors (GPCRs) in the plasma membrane of neurons that are activated by the binding of glutamate, the predominant excitatory neurotransmitter of the central nervous system (CNS). In contrast to the activation of ionotropic glutamate receptors, generating an electrical response on their own^1^, mGluRs evoke responses in the cells via activation of various G-proteins and subsequent signaling cascades^2,3^. mGluRs are obligatory dimers and belong to class C GPCRs. Each subunit contains an N-terminal ligand-binding domain (LBD), a cysteine-rich domain (CRD), and a seven-helix transmembrane domain (TMD). Dimerization of the extracellular domains is required to activate downstream G proteins after glutamate binding, while isolated TM-domains do not spontaneously dimerize^4^.

In mammals, eight subunit isoforms, mGluR1-8, have been identified by sequence (Supplementary Fig. 1) and they are assigned to either group I, II, or III. Generally, group I mGluRs are coupled to a G_q_ protein cascade, whereas group II/III are coupled to a G_i/o_ protein cascade^2,3^. As a result, a group I receptors (mGluR1, mGluR5) stimulate phospholipase C and adenylyl cyclase as well as MAP-kinase^2^. Their location is predominantly postsynaptic. mGluR1 and mGluR5 are widespread in neurons of the CNS^5–9^ but are found also in peripheral nociceptors^10–12^. Group II receptors (mGluR2, mGluR3) are functionally antagonistic to the group I receptors by inhibiting the adenylyl cyclase, and, in addition, activate K^+^ and inhibit Ca^2+^ channels^2^. They are also widespread in neurons, including neurons involved in sensing and conducting pain^13,14^, and are preferentially located in the presynaptic but also in the postsynaptic membrane. Group III receptors (mGluR6, mGluR4, mGluR7, mGluR8) are functionally synergistic to group II receptors, but their location and function are more diverse^2^. They can be found throughout the peripheral and central nervous system. Like group II receptors, mGluR4, mGluR7, and mGluR8 are significantly expressed in pain neurons^15–18^, and their preferential location is also in the presynaptic membrane. mGluR6 is exceptional in both location and function. It is restricted to the retina, is found in the postsynaptic membrane, and operates by stimulating a cGMP phosphodiesterase^2,19^.

Regarding activation of mGluRs, spectroscopic and biochemical approaches revealed an intersubunit reorientation of both the extracellular and transmembrane domains^20–26^. In a recent study, the inclusion of highly resolved structural results on both the apo and a holo conformation provided insight into the activation of mGluR5:^27^ Glutamate, and other orthosteric agonists, promote compaction of the VFT conformation. This signal propagates along the CRDs, sampling at least 4 conformations^28^, to the 7TM domains, moving them closer together, and rotating them by about 20^o^^27^. The TM6-mediated interfaces reorient and generate competence for signaling^29–31^. The use of selective ligands with photoswitchable tethered agonists revealed pronounced cooperativity in the activation process of mGluR2^32^. For group II mGluRs, the interaction between orthosteric (VLC) and allosteric (within the TM) binding sites were analyzed in detail^33,34^.

The dynamics within the receptor start with sub-millisecond LBD dynamics^21^, leading to initial intersubunit rearrangements^35^ in the order of one millisecond^36^, reported for mGluR1, and activation of downstream signaling in the second to minutes range. The kinetics of the receptor dynamics determines, together with its localization, the temporal quality of signals the receptor is sensitive to, ranging from individual synaptic activities^37^ to an integrated signal of local glutamic releasing activity. Therefore, knowledge of the activation and deactivation kinetics might aid to understand subtype functions.

In contrast to GABA B receptors, another class C GPCR which is an obligatory heterodimer, mGluR subunits are reported to form both homo- and heterodimeric receptors. For a few heterodimers, the specific function has been demonstrated: Within-group II, heteromerization has been described for mGluR2/3 by using a single-molecule approach^32^. The data reveal defined interactions between both LBDs. Another well-elaborated example for functional heterodimers is the interaction of the subunits in mGluR2/7^38^. In this heterodimer, the mGluR2 subunit brings the unusually high *EC*_50_ value of mGluR7 not only into the physiological range but leads even to a more efficient activation than in homomeric mGluR2. Evidence for the formation of functional heterodimers has been reported for mGluR2/4^39,40^. This dimer was recently shown to be functionally expressed widely throughout the brain^41^. Dimerization between mGluR1/5 was shown to be present in neurons. When expressed in heterologous cells, heterodimers show intermediate signaling efficacy and a mixed kinetic profile that cannot be distinguished from mGluR1 and mGluR5 responses^42,43^.

In an attempt to gain more systematic insight into the assembly of heterodimers among mGluRs, all combinations of mGluRs, apart from mGluR6, were labeled specifically with the SNAP and CLIP technology and studied by time-resolved FRET^44^. 11 out of the 21 combinations were identified to form heterodimers, including the above examples of mGluR2/3, mGluR2/7 and mGluR2/4. However, the study provided only information about an assembly of subunits to heterodimers, except for mGluR2/4 for which they also proved functional interaction. Recently dimerization propensities among the different mGluR subunits were studied by another fluorescence-based approach and high-efficiency heterodimerization was found within group II as well as weaker heterodimerization between group I (mGluR1) and II (mGluR3)^45^. Notably, mGluR2/3 heterodimers were shown to form with similar or even better efficiency compared to their respective homodimers^45^. Moreover, evidence for the dimerization between mGluR1 (group I) and mGluR3 (group II) was presented, even though it is much weaker than for homodimerization. This differs from previous results in which dimerization between subunits of group I and group II was not observed^44^.

Herein, we investigated all possible heterodimers of the subunits mGluR1-8 both glutamate-induced kinetics and the subunit assembly at the level of the receptors. The kinetics, reporting conformational changes between the two subunits, were monitored by time-dependent FRET changes whereas the assembly was judged by donor dequenching upon acceptor photobleaching. Thus, the ligand-induced FRET changes are assumed to represent a readout for ‘activation’ or on-kinetics and ‘deactivation’ or off-kinetics of the receptors upon fast ligand application and wash, respectively, being aware that the introduced fluorescent proteins prevent downstream signaling.

## Results

### Rationale of the experimental approach

The amino acid alignment (Supplementary Fig. 1) and the degree of sequence homology (Supplementary Fig. 2 and Supplementary Fig. 13) reveal a high degree of homology of 61.8–75.0% within each mGluR group and 39.4–46.4% between the members of different groups. These degrees of homology led us to include the possibility that all eight subunits assemble into functional heterodimers. To investigate this, the subunits of all eight mGluRs were specifically labeled by incorporating either the cyan (CFP) or the yellow (YFP) fluorescent protein in the intracellular i2 loop^35,36^ and the receptors were co-expressed in *Xenopus* oocytes to high expression levels (Methods and Supplementary Fig. 3, Supplementary Data 6). To optimize the signal-to-noise ratio we used the GABA_B_-based quality control-system consisting of an ER-retention signal (GABA_B1_) and a masking sequence (GABA_B2_) at the C-terminus, as described previously^46–49^. This ensures that donor-containing constructs in the membrane are the desired dimers with the acceptor constructs by retaining donor/donor dimers (GABA_B1_/GABA_B1_) in the ER, thereby generating also an enhanced apparent FRET-efficiency (Supplementary Fig. 4).

**Fig. 1:**
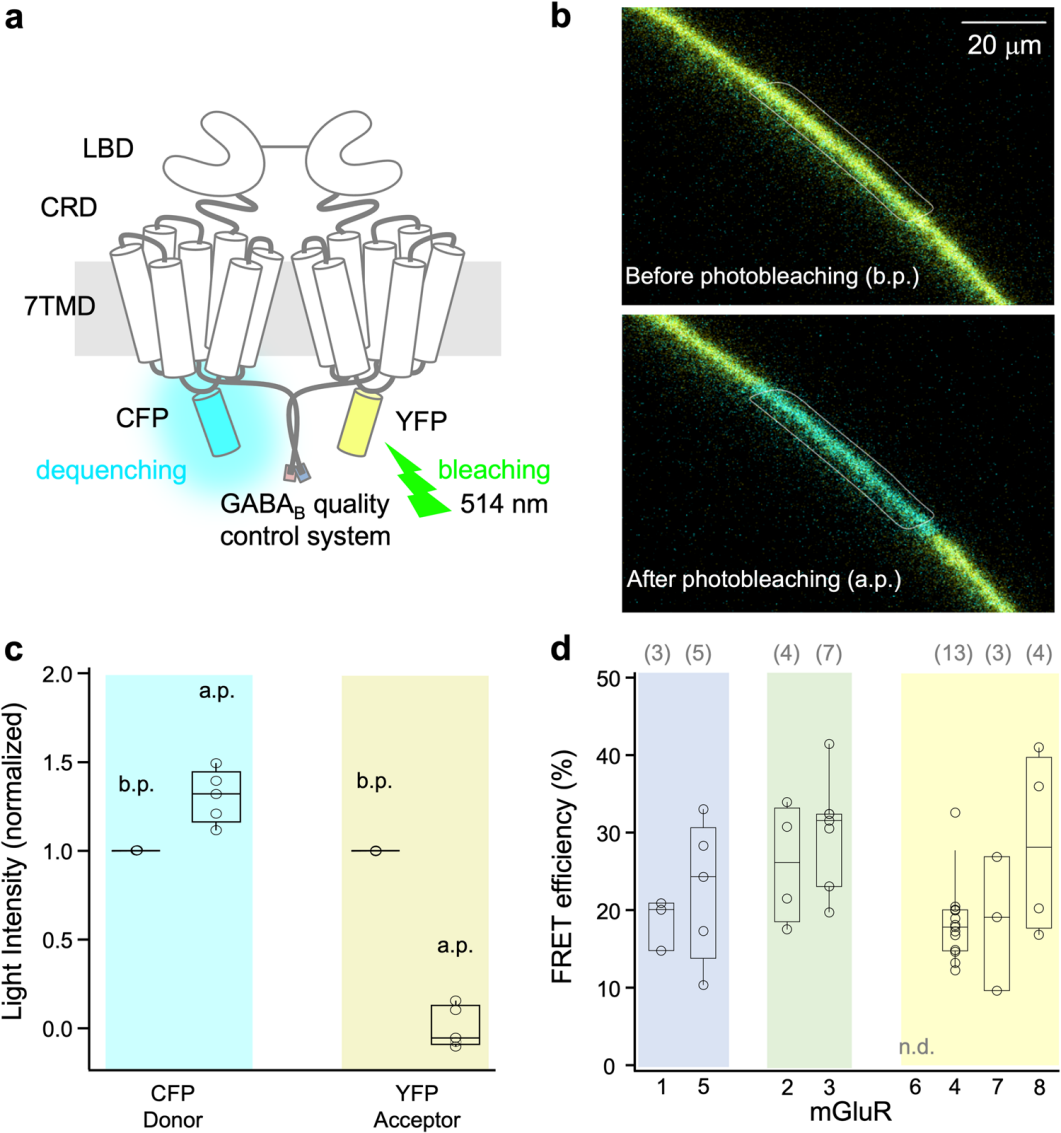
Donor dequenching after photobleaching the acceptor in homodimeric mGluRs. **a** Cartoon of a dimeric mGluR. Each subunit contains an N-terminal ligand-binding domain (LBD) that is connected to a cysteine-rich domain (CRD) which is connected to a seven-helix transmembrane domain (7TMD). The cyan or yellow fluorescent protein, CFP, and YFP are included in the intracellular i2 loop. The quality control system of the GABA_B_ receptor was used to control the composition of the receptors to be analyzed (see Methods). The principle of acceptor-bleaching induced donor dequenching is indicated. **b** Confocal micrograph of an oocyte membrane expressing mGluR2/7 before (top) and after photobleaching with the light of 514 nm (bottom). The membrane region with the bleached yellow signal is easily visible. **c** FRET efficiency for the representative example mGluR5 for donor dequenching by photobleaching of the acceptor. b.p., and a.p. means before and after photobleaching, respectively. The light intensity of the donor was increased by 23 ± 4%. **d** FRET efficiency of the donor was evoked by photobleaching the acceptor for the eight homodimeric mGluRs. The values were obtained from 3 to 7 cells. n.d., not determinable. The numbers of experiments are shown at the top in brackets.

**Fig. 2:**
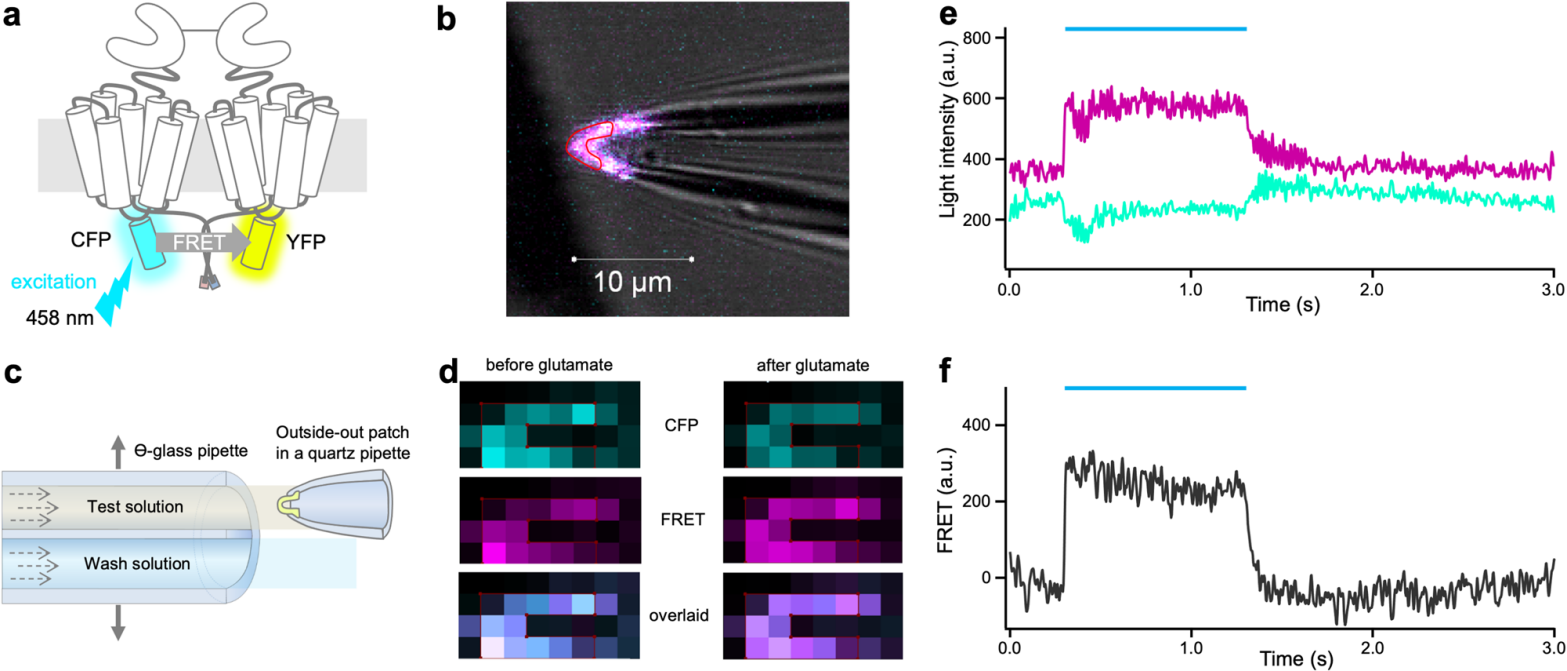
Kinetic FRET measurements in outside-out patches. **a** Cartoon illustrating the principle of FRET measurements by exciting the donor CFP at 458 nm and detecting the emissions of CFP and YFP. **b** Tip of a patch pipette carrying an outside-out patch containing a large number of labeled homodimeric mGluR1. **c** Scheme illustrating fast solution switches at the outside-out patch. A double-barreled Θ-glass pipette delivering two laminar streams of solutions is stepped by a piezo device. **d** Confocal images of a patch before and after adding glutamate. Left panels show signals coming from CFP, FRET, and overlaid channels before adding glutamate. The right panels show a decreased CFP signal, increased FRET signal, and a clear signal change in the overlaid channel, presumably due to a conformational change. **e** Fluorescence signal and glutamate-induced changes in time-dependent traces of YFP (magenta) and CFP (cyan) after correction for crosstalk and photobleaching. The light blue bar indicates the application of glutamate (1 mM). **f** Calculated FRET signal from **e**.

The subunit assembly into dimers was determined in whole oocytes expressing the receptors by photobleaching the acceptor, YFP, and measuring the dequenching of the donor, CFP (Fig. 1a, b; Methods). In contrast, the functional interaction of the subunits was quantified by analyzing time-dependent FRET signals (Fig. 2a) in outside-out membrane patches (Fig. 2b) using confocal patch-clamp fluorometry^50–52^. To this end, the patches were subjected to glutamate jumps from zero to the saturating concentration of 1 mM (10 mM for mGluR7) and back to zero by a piezo switch and two laminar solution flows at the outlet of a Θ-glass pipette (Fig. 2c). These concentration jumps resulted in time-dependent fluorescence changes of CFP and YFP (Fig. 2d, e, Supplementary Data 2) and calculated FRET (Fig. 2f, Supplementary Data 2), mirroring presumably activation and deactivation, respectively. These time courses were quantified by Eqs. (2–6) yielding the time constants *τ*_on_ and *τ*_off_, respectively. This approach was employed recently to study the activation of homomeric mGluR1^36^. With this double strategy, donor dequenching, and receptor kinetics, we analyzed all possible 36 homo- and heterodimeric combinations of human mGluR1-8.

### Homodimeric glutamate receptors mGluR1-8

We first consider donor dequenching in homodimers. For seven of the eight homodimers, the apparent FRET efficiency, shown representatively for mGluR5 in Fig. 1c (Supplementary Data 1), yielded similar values between 18% and 30% (Fig. 1d, Supplementary Data 1), suggesting the formation of homodimers. For mGluR6 the expression was too low for evaluation. To rule out influences on dimerization or detection of fringe fractions due to the GABA_B_-system, we performed control measurements with constructs lacking the GABA_B_ sequences with homodimers from each group (I: mGluR1/1; II: mGluR2/2; III: mGluR4/4). As expected, the apparent FRET overall donors in the region of interest (ROI) decreased, whereas kinetics remains similar (Supplementary Fig. 4 and 5). Without the GABA_B_ system, the donor dimers can also reach the membrane, thus also contributing to the observed average value, suggesting a helpful effect of the GABA_B_ system. Because the resolution of optical microscopy is limited, the contribution of intracellular vesicles near the membrane, containing e.g. donor/donor dimers, cannot be ruled out. Thus, the values represent at best a lower estimate of the FRET- efficiency. In fact, FRET efficiencies estimated from isolated membrane patches are substantially higher (Supplementary Figs. 4 and 11).

**Fig. 3:**
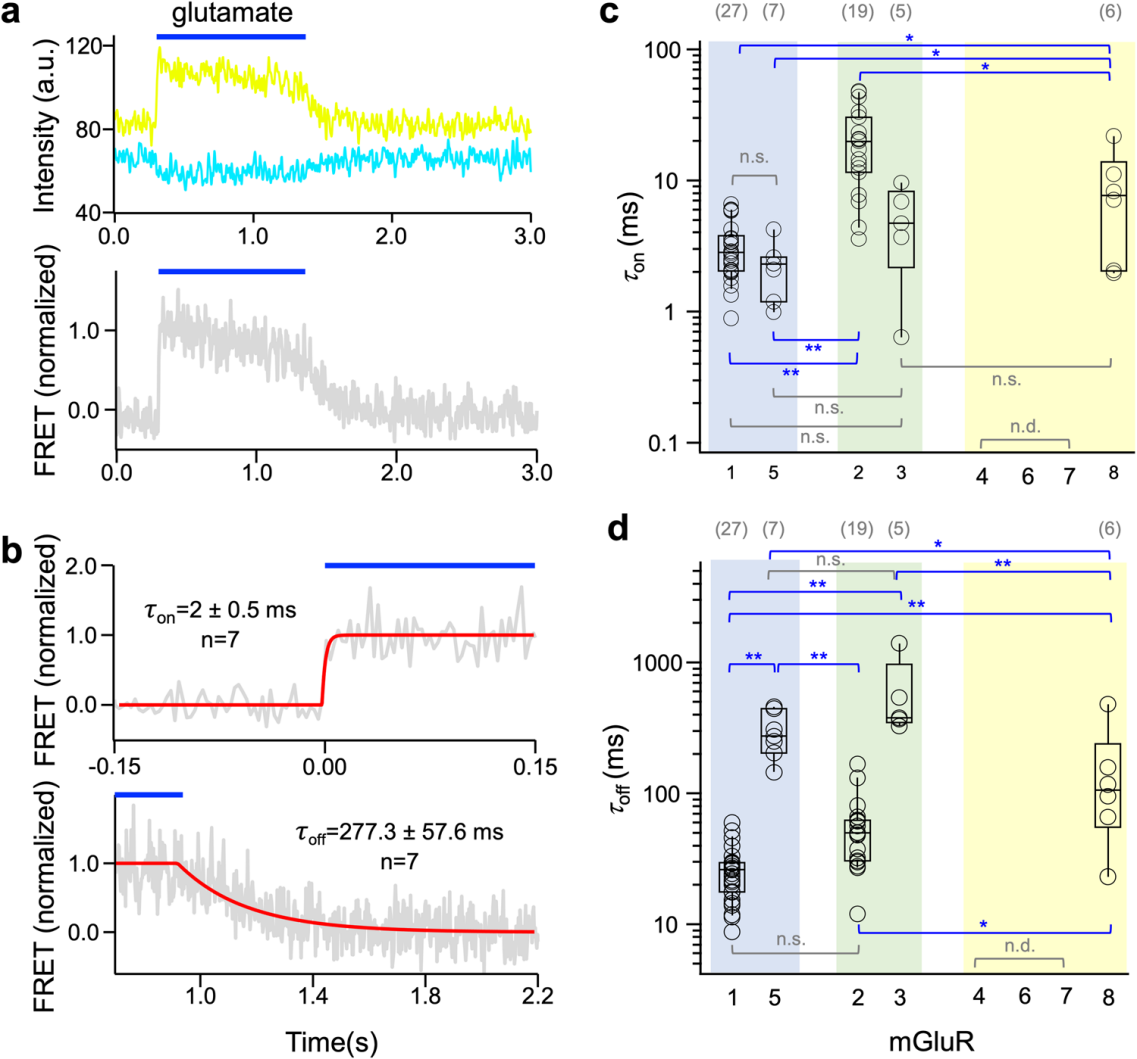
Activation and deactivation kinetics in homodimeric mGluRs. **a** Time course of fluorescence changes for donor and acceptor in dimeric mGluR5 (top) and resulting FRET signal (bottom). Here and in the following, the blue bar indicates the application of 1 mM glutamate. Shown are representative photobleaching and cross-talk corrected traces of CFP (cyan) and YFP (yellow) as well as the corrected normalized FRET signal (gray). **b** Time courses of activation (top, *τ*_on_) and deactivation (bottom, *τ*_off_) of an individual experiment (gray). The superimposed curves are best fits to average traces obtained from *n* = 7 individual traces each. **c**, **d** Activation (**c**) and deactivation (**d**) time constants for the mGluRs. Three mGluRs did not show evaluable time courses. n.d., not determinable. Significant differences are indicated (ANOVA followed by Turkey-Kramer posthoc test, see Methods: *p < 0.05, **p < 0.01). In c and d the numbers of experiments are shown at the top in brackets.

When considering the time courses of activation and deactivation reported by FRET, as shown representatively for mGluR5 in Fig. 3a, b, sufficiently robust signals could be obtained from mGluR1, mGluR5, mGluR2, mGluR3, and mGluR8. Individual traces were subjected to a fit with an exponential function, and fit results of at least four traces were averaged (see Supplementary Fig. 6 (strategy), 7a (individual fits), and 8 (all fit results) as well as Supplementary Tables 2 and 3 for results and statistics). This means that members of all three groups evoke conformational changes leading to time-dependent FRET signals, suggesting that these homodimers are functional. In contrast, mGluR4 and mGluR7 did not provide ligand-dependent FRET signals, indicative of either no glutamate-induced interaction or of different conformational changes not generating a FRET signal.

Among the five homodimers generating FRET-signals, considering *τ*_on_ and *τ*_off_ reveals marked and characteristic differences: *τ*_on_ for the group I members mGluR1 and mGluR5 (Fig. 3a, b, Supplementary Data 3) and group II member mGluR3 are rapid and at the border of our resolution (Fig. 3c, Supplementary Data 3, Supplementary Fig. 8a), whereas for the group II member mGluR2 as well as for the group III member mGluR8 it is significantly slower (Fig. 3c). As reported earlier for mGluR1^36^, *τ*_off_ is slower than *τ*_on_ in all five homodimers (Fig. 3d, Supplementary Data 3). Deactivation kinetics, *τ*_off_, strongly differs within group I and within group II, with mGluR5 being slower than mGluR1 and mGluR3 being slower than mGluR2. In the only measurable group III member mGluR8, the *τ*_off_ value is in-between the fast and slow deactivating isoforms of the other groups. These results show that there is a characteristic kinetic pattern that can be used for investigating the role of the individual subunits in heterodimers.

We also tested the influence of the GABA_B_-system on kinetic measurements by omitting it for the example mGluR1/1, as it delivers data with the highest quality. Also, here no alteration of the receptor kinetics by the GABA_B_ sequences was observed whereas the apparent FRET was expectedly reduced (Supplementary Figs. 4 and 5).

### Heterodimeric glutamate receptors

We first tested heteromerization by measuring the apparent FRET efficiency by donor dequenching upon acceptor photobleaching in whole oocytes (Supplementary Fig. 9). 16 out of 28 heterodimers provided a detectable FRET change. The values of the heterodimers are in the same order as those of the homodimers but cover a slightly wider range from 13% for mGluR4/8 to 35% for three heterodimers of mGlu2, namely mGluR2/3, mGluR2/4, mGluR2/8. Noticeable is also, that mGluR2 produces FRET with all other mGluRs whereas mGluR6 produces FRET only with mGluR2, and does even not express as a homodimer.

**Fig. 4:**
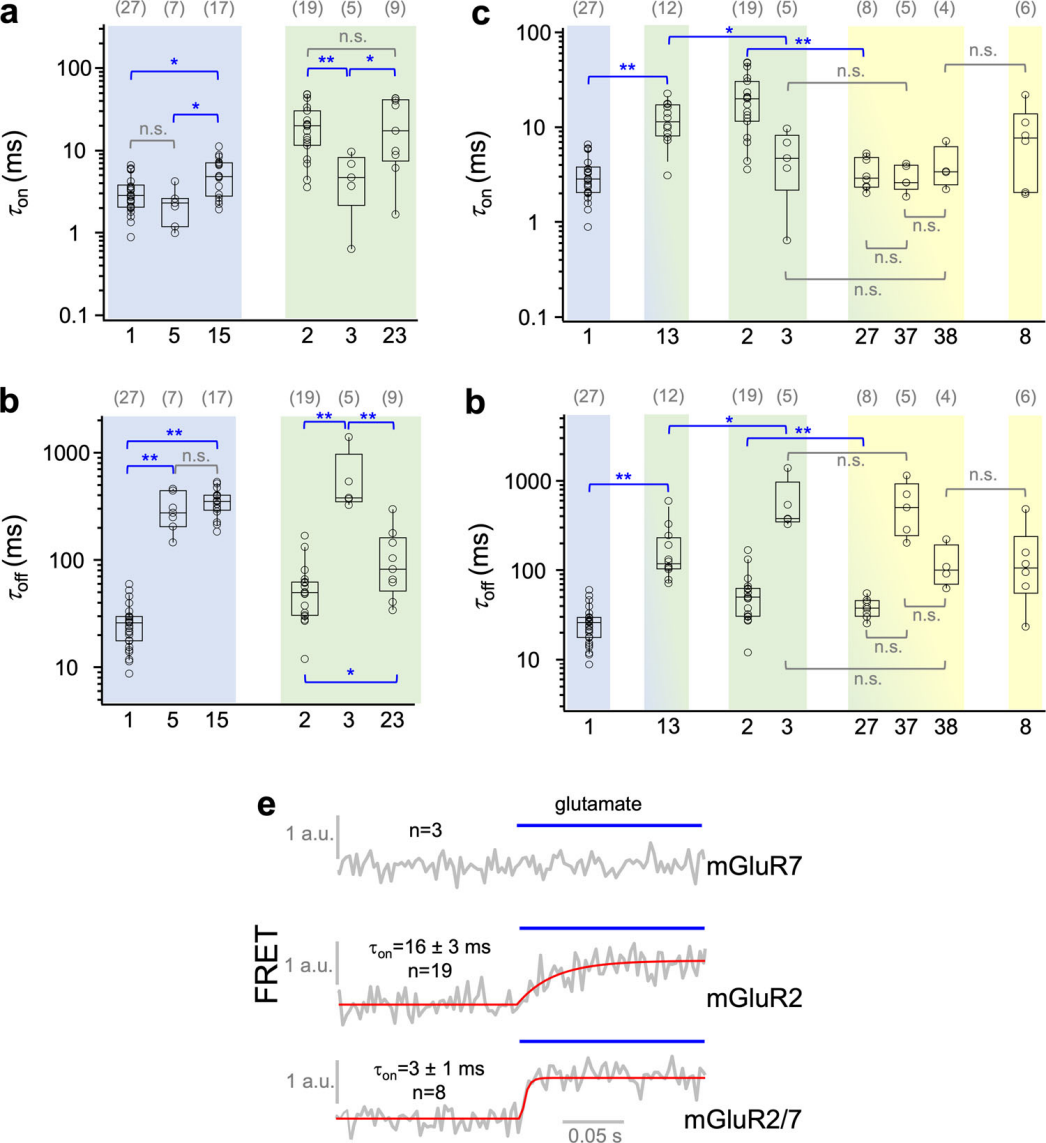
Kinetic responses of heterodimeric mGluRs. **a**,**b** *τ*_on_ and *τ*_off_ for two heterodimers in groups I and II. **c**, **d** *τ*_on_ and *τ*_off_ for two heterodimers between group I and II and three heterodimers between group II and III. Homomeric data from Fig. 3 are shown for comparison. Significant differences are indicated (ANOVA followed by Turkey-Kramer posthoc test, see Methods: *p < 0.05, **p < 0.01). The computed *p*-values are provided in Supplementary Table 3. n.s. indicates that the difference is ‘not significant’. **e** Averaged time traces obtained with 1 mM glutamate for mGluR7 (*n* = 3), mGluR2 (*n* = 19), and mGluR2/7 (*n* = 8) with fitted exponential functions (red curves). mGluR7 is a strong accelerator in mGluR2/7 compared to mGluR2. In **a**–**d**, the numbers of experiments are shown at the top in brackets.

We then tested the glutamate-induced conformational rearrangements between the subunits in heterodimers by our kinetic FRET approach (for fitted traces see Supplementary Fig. 7a–d, Supplementary Fig. 8, and Supplementary Tables 2 and 3). Within the groups, only mGluR1/5 in group I and mGluR2/3 in group II provided evaluable kinetic signals (Fig. 4a, b, Supplementary Data 4). While activation in mGluR1/5 is somewhat slow compared to the respective homodimers, mGluR2/3 is as slow as the slower subunit mGluR2 (Fig. 4a). Regarding deactivation within groups I and II, the slower subunit mGluR5 dominates in mGluR1/5 while in mGluR2/3 the speed is intermediate between mGluR2 and mGluR3.

Between the groups, we could analyze the kinetics from one heterodimer between group I and II and three heterodimers between group II and III, but from no heterodimer between group I and III. Activation of mGluR1/3 is slower than that of the respective homodimers. In contrast, a remarkable property was observed for mGluR7: While homodimeric mGluR7 did not yield any evaluable kinetic response (tested with up to 10 mM glutamate), it accelerated activation in the heterodimers mGluR2/7, as compared to homodimeric mGluR2, by nearly an order of magnitude (Fig. 4c, e, Supplementary Data 4).

Regarding deactivation kinetics of heterodimers within groups, mGluR1/5 (group I) showed the kinetics of the slower subunit, while mGluR2/3 (group II) showed intermediate kinetics of the respective homomers. Similarly, group I/II heteromer mGluR1/3 showed intermediate kinetics compared to the homomers. All heteromers between groups II and III showed the kinetics of the faster subunits (when regarding the lack of mGluR7 activation as infinitely slow activation) (Fig. 4d, Supplementary Data 4).

**Fig. 5:**
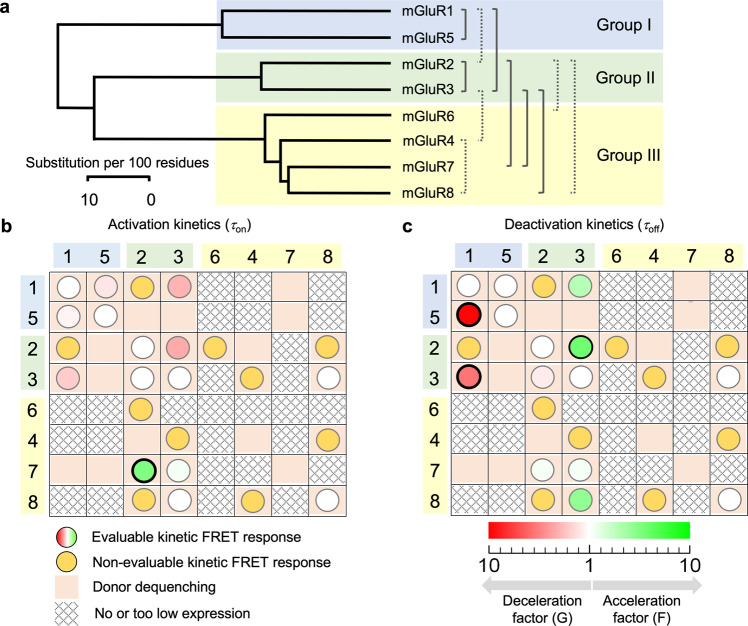
Overview of the specific functional interactions in heterodimeric mGluRs. **a** Phylogenetic tree with seven functional interactions specified by activation and deactivation time constants (solid brackets) and four functional interactions identified by smaller, kinetically not quantifiable responses (Stippled brackets). **b** Matrix summarizing relative activation kinetics (circles) of the heterodimers among the eight subunits mGluR1-mGluR8 with respect to the homodimers and donor dequenching (salmon fields). The amount of the kinetic effects, within each column, are color-coded according to the scale in c, providing a factor of acceleration (*F*, green) and deceleration (*G*, red) with respect to the homodimers. For example, the intensive green circle in row 7 column 3 reads that activation in mGluR2/7 is strongly accelerated with respect to mGluR2 by the color-coded factor F. Significant differences are indicated by a black bold rim of the circles. White circles indicate an equal time course to the homodimer of the column. The main diagonal contains the properties of the homodimers. **c** Matrix summarizing deactivation kinetics of the heterodimers. Same symbols as in **b**.

In addition to the seven glutamate-responsive heterodimers described so far, five other ones, mGluR1/2, mGluR2/6, mGluR2/8, mGluR3/4, and mGlu4/8, provided time-dependent responses (Supplementary Fig. 10), which could, however, not be quantified kinetically due to insufficient amplitudes of the signals (Supplementary Figs. 11 and 15). This could be a result of both, low expression (Supplementary Fig. 3, Supplementary Data 6) or only small FRET changes outside the group I (Supplementary Figs. 9 and 15). Again, three of these four heterodimers contain one subunit from group II but with mGluR4/8 also a heterodimer within group III is included, indicating that glutamate-responsive heterodimerization is possible within all three groups (Fig. 5a, Supplementary Data 5). Despite the lower resolution of the signals from these four additional heterodimers, they prove glutamate-induced conformational changes. It is noteworthy that analyzing the amplitude changes in donor and acceptor time traces from the patch-derived signals allows us to quantify the resting FRET efficiency (see Methods) as well as glutamate-induced changes in FRET efficiency, without tedious calibrations of quantum yields and detection efficiencies. Further, disturbing contributions of ER-vesicles containing donor: donor dimers near the membrane are minimized by patch excision. As a result, the observed FRET efficiencies (Supplementary Fig. 4) are higher than the values from whole oocytes.

To exclude that our results on heterodimers depend on the particular combination of the fluorescence label (CFP or YFP) and the GABA_B_ sequence of the quality control system (C1 and C2) (see Methods)^49^, we tested for the examples mGluR1/5 and mGluR1/3 the opposite combination by swapping both the fluorescence labels and the GABA_B_ sequence (Supplementary Fig. 12, Supplementary Data 6). A similar test was done also for dequenching analysis. For the key examples mGluR1/2, mGluR1/3, mGluR2/4, and mGluR1/5, the fluorophores were swapped and the result for FRET efficiency was in the same range (Supplementary Fig. 14). The result was that neither activation nor deactivation kinetics are different for the two combinations, supporting the notion that for the tested example mGluRs the observed kinetics are indeed properties of the activation machinery of the specific heterodimeric receptors.

Furthermore, to rule out relevant effects of the GABA_B_ quality control system on dimerization in heterodimers, we performed in analogy to homodimers control measurements with constructs lacking the GABA_B_-sequences, using mGluR1/2, and mGluR2/4, and mGluR1/4. As with constructs containing the GABA_B_ sequence, for mGluR1/2, and mGluR2/4 the apparent FRET of the donors decreased, in both excised patches and the ROI of oocytes (Supplementary Fig. 4), as FRET inactive donor: donor dimers now contribute to the signal. In contrast to these heterodimers, but in accordance with the constructs containing the GABA_B_ sequence, for mGluR1/4 lacking the GABA_B_ sequence FRET was not observed. Thus, the lack of FRET in mGluR1/4 was not caused by an artificial ER-retention due to a failing GABA_B1_/GABA_B2_ interaction.

## Discussion

Herein, the power of confocal patch-clamp fluorometry^50,51^ was used to systematically screen glutamate-induced conformational changes of all eight mGluR subunits in heterodimers by kinetic analyses. Regarding the type of constructs used herein, we like to note first that the primary dimer interface in mGluRs is the extracellular LBD^32^ whereas the interventions in our subunit constructs are located on the intracellular side. Crystal structures have shown that glutamate binding shortens the distance between the C-termini of the LBDs in the extracellular domain^23^. Regarding the time scale of the related conformational changes, single-molecule studies on the dynamics of the LBD/VFT-domain^21,22^ revealed sub-millisecond dynamics on the level of the LBDs. It has been postulated that the two transmembrane regions of the subunits approach each other upon rearrangement of the extracellular domains^23^. This has been recently verified by cryo-EM and crystallography data on mGluR5^27^, mGluR2^30,31^, mGluR3^53^, mGluR1^54^ and mGluR2/7^31^. In the present study, we analyzed inter-domain movements in all mGluRs with labels at the intracellular loops 2, assuming that the intramolecular mechanics of activation is largely preserved. Both distance changes and twisting motions, affecting the relative fluorophore orientation and thus the orientation factor *κ*^2^ and thus the Förster radius *R*_*0*_, will result in a FRET-change. These influences are not distinguished here. Similar approaches to determine the kinetics of mGluR1 were used previously^35,36,55^.

Our results show that at least 11 out of 28 possible heterodimers form receptors that undergo conformational changes upon glutamate binding (Fig. 5a). Glutamate-responsive heterodimers were identified within groups I, II, and III and between groups I and II as well as between group II and III subunits, but not between group I and III subunits. The results are summarized in two matrices in which identified kinetic properties are indicated by circles (Fig. 5b, c). The five circles in the main diagonals indicate the glutamate-responsive homodimers. The relative acceleration and deceleration of on- and off-kinetics, termed here activation and deactivation, respectively, with respect to the homodimer of the actual column, are encoded by colors (red-white-green). Green indicates the acceleration factor F whereas red a deceleration factor G. For example, in Fig. 5b the intensive red circle in row 4 column 1 reads that activation in mGluR1/3 is strongly decelerated with respect to mGluR1 by the color-coded factor *G*. The bold blue margin indicates a significant difference. The ochre circles indicate a clear kinetic response but the time courses could not be evaluated because of an insufficient signal-to-noise level.

One of the most striking examples we identified herein is the strong accelerating effect of activation for mGluR7 in mGluR2/7 activation while deactivation of mGluR2/7, as well as activation and deactivation in mGluR3/7, are not affected (Fig. 4c, e). Interestingly, it was reported that mGluR7 will also increase the *EC*_50_ in heteromers^38^. Another prominent feature of activation of the heterodimers (Fig. 5b) is the strongly decelerated kinetics in mGluR1/5 and mGluR1/3 with respect to the corresponding monomers. For deactivation, a variety of effects was observed: deceleration to the slower kinetics of the subunits in mGluR1/5 (group I), intermediate kinetics in mGluR2/3 and mGluR1/3 (group II), as well as acceleration to the kinetics of the faster subunit in mGluR2/7, mGluR3/7, and mGluR3/8 (all involving a group III isoform). In no case, deactivation was outside the range covered by the deactivation kinetics of individual subunits. Figure 5b, c also include information about donor dequenching, indicating that in total 16 heterodimers assemble. Hence, 5 of the heterodimers producing dequenching did not respond to glutamate under our experimental conditions. Notably, this does neither rule out small or very slow conformational changes nor conformational changes providing no detectable FRET change.

This argument also likely applies to heteromers reported by Doumazane et al.^44^, but not detected here. Our data agree with those in this study regarding the lack of observed group I/group III heterodimers. However, we did detect group I/group II hetero-dimers not reported by Doumazane et al.^44^ or in another recent study focused on LBD interactions^45^. One difference between these studies and our study is our use of constructs with truncated C-termini instead of full-length constructs. It was reported that the C-terminus can strongly modulate the formation and trafficking of mGluR heteromers even between alternative spliced forms of the same isoform^56–58^. This modulation is mediated by an ER-retention signal and its masking is similar to the ones in GABA_B_. Alternative splice products are reported for the majority of GPCRs^2^, often forming different C-termini. Thus, our choice to omit the C-terminus might enable the observation of heteromers otherwise only formed between permissive splice forms.

Considering the heterodimer mGluR2/4, a relevant difference in receptor function appeared between our results and a previous study by Yin and coworkers^40^. While our biophysical approach did not yield FRET signals upon glutamate application (Fig. 5b, c), Yin and coworkers described signal transduction in neurons induced by mGluR2/4 receptors. These findings were extended by Meng et al. showing functional expression throughout the brain^41^. Again, possible reasons for a missing FRET change in our study are a poor signal-to-noise ratio, due to low expression, or a conformational change generating only low or even no FRET changes due to an unfortunate geometry.

While most of the observed activation kinetics (*τ*_on_) are close to the temporal resolution of the experiment, the deactivation kinetics (*τ*_off_) varied widely over more than an order magnitude. For a pure binding reaction, one could assume a diffusion-limited binding and an unbinding rate determining both the affinity and unbinding kinetics. However, there is no correlation between the reported *EC*_50_ (Supplementary Tables 1 and 3) and the observed *τ*_off_. This is consistent with the activation and deactivation of mGluRs, which are complex coupled reactions passing through sets of several (conformational) states as extensively reported^15,27,28,33,38,55^. In our previous work^36^, we observed a concentration dependency of *τ*_on_ which vanished at high concentrations when intracellular rearrangements are slower than the diffusion-limited binding. In contrast, *τ*_off_ cannot be influenced by binding, as no free ligand is available after the jump back to wash solution. Concentration-dependent *τ*_off_ could only arise from binding sites not occupied at lower concentrations. As we are using concentrations exceeding the *EC*_50_ by at least 1.5 orders of magnitude (except for mGluR7; see Supplementary Table 1), this is unlikely for the experiments reported here.

Recently a systematic study investigated deactivation mechanisms of mGluR subtypes^59^. Strikingly, their findings on which subtypes undergo internalization upon glutamate exposure coincide with our findings: all subtypes showing slow deactivation also showed glutamate-induced internalization: mGluR5 within the group I, mGluR 3 within group II, and mGluR7, and mGluR8 in group III. Only mGluR1 showed glutamate-induced internalization in a report by Abreu and coworkers^59^ and fast deactivation in this and previous studies^36^. While this correlation is consistent with cells ensuring a fast signal adaptation upon stimulation, conclusions on the functional relevance of this finding are beyond the scope of this study. However, one is tempted to hypothesize that slow inactivating heterodimers (mGluR1/5, mGluR1/3, mGluR3/7, and mGluR3/8) might also show glutamate-dependent internalization. Note that our kinetic data, due to the isolated nature of the native membrane within the patch, are likely not affected by any internalization, phosphorylation, or other process but represent the pure receptor dynamics. Possible links between sequences and dimerization propensity are discussed in Supplementary discussion and Supplementary Fig. 11.

In conclusion, group II subunits have a higher propensity to form functional heterodimers with group I and group III subunits than group I with group III subunits. This differential propensity might play an essential role in multiple functions in neurons, potentially both at the pre-and postsynaptic membrane. Our results, therefore, suggest further targets for the analysis of heterodimers in native cells.

## Methods

### Generation of mGluR FRET-sensors

Based on the previously described plasmids for the *E* sensor in rat mGluR1^29^ *E* sensors for all human mGluR subtypes with the complete N-terminal sequence were constructed in a pGEM-HEnew vector. The coding sequences for human mGluR1-8 (ACC#NM_000838, ACC#NM_000839, ACC#NM_000840, ACC#NM_000841, ACC#NM_000842, ACC#NM_000843, ACC#NM_000844, ACC#NM_000845) were provided by GenScript or cDNA resource Center in pcDNA3.1 derivatives and used as templates. Based on an alignment with PROMALS3D, YFP (AGM20711.1) or CFP (AGM20712.1) were integrated into the intracellular loop i2 behind the conserved RI residues (green vertical line in Supplementary Fig. 1) using introduced Nhe1 and Xho1 restriction sites. To ensure the heterodimerization between YFP and CFP labeled subunits resulting in the *E* sensor C-terminal tails of the GABA_B_ receptor subunit 1 (CAA71398:1) in CFP constructs and subunit 2 (AAD03335.1) in YFP constructs were incorporated by replacing mGluR tails using overlapping PCR. The correctness of the plasmids was checked by restriction analysis and sequencing (Microsynth SEQLAB, Göttingen, Germany). cRNA for injection was prepared using the mMESSAGE mMACHINE T7 kit (Ambion Inc, Austin, USA).

### Oocyte Preparation and cRNA injection

Oocytes of *Xenopus laevis* were obtained either from Ecocyte® (Castrop-Rauxel, Germany) or were harvested surgically from female adults under anesthesia (0.3% 3–aminobenzoic acid ethyl ester) as reported previously^60^. The procedures had approval from the authorized animal ethics committee of the Friedrich Schiller University Jena. To achieve high expression, 20–40 ng of the respective cRNAs were injected into the oocytes as a 1:1 stoichiometric mixture. The oocytes were incubated at 18 °C and used between 4 and 6 days after injection.

### FRET estimation with acceptor photo bleaching

Expression of the sensors was evaluated with fluorescence microscopy as described below. Basal FRET, confirming dimer formation, was estimated by iteratively photo bleaching the acceptor in a membrane region with a 514 nm laser until remaining signals were stable. FRET efficiency was estimated as

1 $${E}_{f}=\left(1-\frac{{D}_{{DA}}}{{D}_{D}}\right)$$

with *D*_*DA*_ and *D*_*D*_ being the donor signal before and after photo bleaching, respectively. Control experiments with only acceptor constructs confirmed that no YFP up-conversion^61^ distorts the observed *FRET* under the conditions used. Oocytes with expression of both CFP and YFP were selected for patch experiments.

### Patch formation and solution application

Outside-out patches from *Xenopus* oocytes were obtained by using a standard patch-clamp technique^62^. The patch pipettes were pulled from quartz tubing (P-2000, Sutter Instrument, Novato, USA) with an outer and inner diameter of 1.0 and 0.7 mm, respectively (VITROCOM, New Jersey, USA). The corresponding pipette resistance was 0.9-2.3 MΩ. The bath and pipette solution contained (in mM): 150 KCl, 1 EGTA, and 10 HEPES (pH 7.4 with KOH). The optical recording was carried out at room temperature using an Axopatch 200B amplifier to confirm intact Gigaohm seals of the patches (Axon Instruments, Foster City, CA). Electrophysiology was controlled by the ISO3-Software (MFK Niedernhausen, Germany). A patch pipette with an outside-out patch was moved close to the outlet of a double-barreled Θ-glass pipette (diameter ≈ 100 µm) that was mounted on a piezo actuator (Physik Instrumente, Karlsruhe, Germany). A computer-controlled device stepped the piezo and thus displaced the theta-glass pipette between a position in the wash solution (control) and the test solution containing 1 mM L-glutamate (Sigma Life Science) (Fig. 2c). Blue bars above time traces indicate time intervals of exposure to 1 mM glutamate. The flow speed of the solution was set to ~130 mm/s. The time course of solution exchange was measured by following the fluorescence of a 1 µM DY647 solution in the line scan mode of the microscope. Fitting of such time courses at the patch yielded a mean time constant for the solution exchange of 220 ± 30 μs.

### FRET in outside-out membrane patches

Fluorescence images were recorded through a 40x/1.2 C-Apochromat water-immersion objective with a confocal microscope (LSM710, Carl-Zeiss, Jena). CFP and YFP were excited with the 458 or 514 nm line of an Argon laser implemented in the microscope, and the detection channel was set to 459–508 nm and 517–581 nm, respectively. To maximize the image frequency and signal, small images of 16 × 8 pixels and a wide pinhole (4.7 airy units) were used. Typically, an imaging rate of 357 Hz was achieved. The electrophysiology set-up triggered both the microscope and the piezo device.

Only oocytes with the highest receptor expression were selected to obtain a sufficiently high signal-to-noise ratio. To obtain the best interpretable conditions, we massively overexpressed the subunits. A glutamate concentration of 1 mM was used for most of the experiments, which is much higher than the EC_50_ value known for the subunits (Supplementary Table 1), suggesting that the observed activation kinetics are no longer rate-limited by diffusion and binding as shown for mGluR1 by our group previously^36^.

### Analysis of data from outside-out patches

#### Extraction and correction for photo bleaching

Time series were extracted by selecting regions of interest from the confocal images. Data were analyzed using the Igor Pro 7.0.9.1 software (Wavemetrics®) with an in-house written procedure. Direct excitation of the acceptor (YFP) by light at 458 nm was negligible. Crosstalk of the donor (CFP) signal to the acceptor detection channel was subtracted. Afterwards, signals from both channels were corrected for photo-bleaching as outlined in Supplementary Fig. 6: traces were masked from 50 ms before to 2500 ms (mGluR1 was refitted with masks ending at 850 ms) after the ligand application. The remaining trace was fitted: Donor signals were fitted with a single exponential (*k* = 1; typical τ_bleach1_ = 0.66 s), FRET signals were fitted with the sum of two exponentials with one time-constant fixed on the donor value (*k* = 2; typical τ_bleach2_ = 2.86 s) according to

2 $${{{{{\rm{Signal}}}}}}({{{{{\rm{t}}}}}})={{{{{\rm{offset}}}}}}+\mathop{\sum }\limits_{{{{{{\rm{i}}}}}}=1}^{k}{{{{{{\rm{A}}}}}}}_{{{{{{\rm{i}}}}}}}\times \exp ({-{{{{{\rm{t}}}}}}/{{{{{\rm{bleach}}}}}}}_{{{{{{\rm{i}}}}}}})$$

The fitted bleaching decay was subtracted from the data before further calculations according to

3 $${{{{{\rm{Signalcor}}}}}}({{{{{\rm{t}}}}}})={{{{{\rm{Signal}}}}}}-\mathop{\sum }\limits_{{{{{{\rm{i}}}}}}=1}^{k}{{{{{{\rm{A}}}}}}}_{{{{{{\rm{i}}}}}}}\times \exp ({-{{{{{\rm{t}}}}}}/{{{{{\rm{bleach}}}}}}}_{{{{{{\rm{i}}}}}}})$$

All bleaching correction was manually verified and rejected if needed. Changes in bleaching dynamics due to ligand-altered FRET efficiency were assumed to be negligible.

Corrected FRET was calculated according to

4 $${{{{{{\rm{FRET}}}}}}}_{{{{{{\rm{cor}}}}}}}={{{{{{\rm{Ch}}}}}}}_{{{{{{\rm{FRET}}}}}}}{-}{{{{{\rm{f}}}}}}\times {{{{{{\rm{Ch}}}}}}}_{{{{{{\rm{CFP}}}}}}}$$

Ch_FRET_ and Ch_CFP_ represent the FRET and CFP signals, respectively. f is a correction factor that depends on the quantum yield and detection efficiencies of the donor and acceptor. It was calibrated by minimizing the correlated signal fluctuations generated by slight fluctuations of the pipette position while preserving the anticorrelated signal change due to FRET. This corrects for slight piezo-induced vibrations as well as contributions of dimers with identical fluorophores. Because the level of FRET_Cor_ depends on the expression level and the patch size, only kinetics and not amplitude of the changes in the so corrected trace were considered quantitatively.

#### Kinetics of the glutamate induced FRET-changes

For quantifying the activation time course, an exponential function was fitted to the individual time courses of the experiments evoked by stepping from zero to 1 mM glutamate according to

5 $${{{{{{\rm{FRET}}}}}}}_{{{{{{\rm{cor}}}}}}}({{{{{\rm{t}}}}}})={{{{{\rm{A}}}}}}\ast (1-\exp (-{{{{{\rm{t}}}}}}/{{{{{{\rm{\tau }}}}}}}_{{{{{{\rm{on}}}}}}}))$$

*τ*_on_ is the time constant. To minimize in fits of fast time courses effects of time jitter, generated e.g. by variable flow speed, pipette position, or LSM-trigger jitter, the time point of the signal start, *t*_0_, was included as a fit parameter and the fits were performed with

5a $${{{{{{\rm{FRET}}}}}}}_{{{{{{\rm{cor}}}}}}}({{{{{\rm{t}}}}}})=\left\{\begin{array}{cc}0 \hfill & t < {t}_{0}\\ {{{{{\rm{A}}}}}}\ast (1-\exp (-\,({{{{{\rm{t}}}}}}-{{{{{{\rm{t}}}}}}}_{0})/{\tau }_{{{{{{\rm{on}}}}}}})) & t\ge {t}_{0}\end{array}\right.$$

To avoid blurring of the kinetics signals were aligned on t_0_ before summing. A time interval of −100 ms to +300 ms relative to the jump time into the glutamate was fitted.

To determine the deactivation time constant, *τ*_off_, the normalized time courses, obtained when jumping from 1 mM glutamate to zero, were fitted with

6 $${{{{{{\rm{FRET}}}}}}}_{{{{{{\rm{cor}}}}}}}({{{{{\rm{t}}}}}})={{{{{\rm{A}}}}}}\ast \exp (-{{{{{\rm{t}}}}}}/{\tau }_{off})$$

In fits of fast time courses effects of a time jitter were minimized by including the time point of the signal start, *t*_0_, and using

6a $${{{{{{\rm{FRET}}}}}}}_{{{{{{\rm{cor}}}}}}}({{{{{\rm{t}}}}}})=\left\{\begin{array}{cc}0 \hfill& {{{{{\rm{t}}}}}} < {{{{{{\rm{t}}}}}}}_{0}\\ {{{{{\rm{A}}}}}}\ast \exp (-\,({{{{{\rm{t}}}}}}-{{{{{{\rm{t}}}}}}}_{0})/{\tau }_{{{{{{\rm{off}}}}}}}) & \,{{{{{\rm{t}}}}}}\ge {{{{{{\rm{t}}}}}}}_{0}\end{array}\right.$$

A time interval of −100ms to +1000 ms relative to the jump-time back to wash solution was fitted. (mGluR1 was refitted with −100 to +300 ms). Per patch 1 to 3 concentration jump-experiments were performed, evaluated and positive selected repetitions were summed.

#### Selecting unsuccessful measurements in membrane patches

Selected time-traces and their fits were identified by the following criteria:

1. successful photo-bleaching correction (flat base line)
2. anti-correlated signal changes in donor and FRET-channel (pipette stable in solution flow)
3. converging fit and fit errors <500% (fit not misled by noise)
4. FRET-change amplitude >150% standard deviation of baseline and >10 a.u. (data with sufficient signal-to-noise ratio)
  Remaining experiments were pooled by expressed subtype combination. Within each pool, traces were removed when the following criteria were met:
5. τ_on_ > 5x median(τ_on_): imperfect pipette position in the solution stream
6. τ_off_ > 5x median(τ_off_): i.e. accidental fit of photo bleaching after imperfect photo-bleaching correction
7. τ_off_ < 20x median(τ_off_): i.e. accidental fit of high singe sample signal change in noise

Additionally, τ_on_ and τ_off_ in each pool were tested for outliers using “median absolute deviation (MAD)” as described e.g. in^63^. Pools retaining 3 or more experiments were considered to be successful and further analyzed.

For evaluations, normally distributed values were assumed. For mGluR1, τ_on_ values did not pass a Shapiro-Wilk-test for normal distribution whereas log(τ_on_) did. This is also plausible, as τ_on_ and τ_on_ cannot be negative but a normal distribution covers negative values as well. Thus, all further statistical tests were performed on log(τ_on_) and log(τ_off_), essentially assuming log-normal distributions of τ_on_ and τ_on_. The number of patches that were used in the evaluations and statistics are provided by Supplementary Tables 2 and 3.

#### Estimation of time-dependent FRET efficiency from concentration jump experiments

In the FRET time courses two equilibrium conditions are present: resting (1) and glutamate exposed (2). From the respective donor- and FRET-signals absolute FRET-efficiencies can be estimated according to

7 $${E}_{f1}=\left(\frac{{D}_{2}}{{D}_{1}}-1\right){\left(\frac{{D}_{2}}{{D}_{1}}-\frac{{{FRET}}_{2}}{{{FRET}}_{1}}\right)}^{-1}$$

8 $${E}_{f2}=1-\left(\frac{{D}_{2}}{{D}_{1}}\right)\left(1-{E}_{f1}\right)$$

9 $${\triangle E}_{f}=1-\left(\frac{{D}_{2}}{{D}_{1}}\right)\left(1-{E}_{f1}\right)-{E}_{f1}={E}_{f1}\left(\frac{{{FRET}}_{2}}{{{FRET}}_{1}}-1\right)$$

*E*_*f*_ is the FRET-efficiency, D the donor-channel signal, FRET the acceptor-channel signal after cross-talk and direct excitation correction (also correcting for acceptor:acceptor dimers). The indices describe the respective equilibrium conditions. This approach assumes that all donor fluorophores are in a 1:1 stoichiometry with acceptors. Note that only ratios of signals of the same channel are used, thus different detection efficiencies, quantum yield etc. do not have to be considered. Results are summarized in Supplementary Fig. 15a and potential systematic errors are discussed in Fig. 15b.

### Statistics and reproducibility

All experiments were performed at least three times (for exact numbers of the experiments, data selection and statistics see Supplementary Table 4). Oocytes from at least two different animals were used. For statistical tests log(τ_on_) and log(τ_off_) were considered, as they were normally distributed. Statistical analysis was performed using the Real Statistics Resource Pack software (Release 7.6). Copyright (2013–2021) Charles Zaiontz. www.real-statistics.com within Microsoft Excel®. Error bars represent SEM. Box plots include all individual data points, the median, the 25–75% interval as boxes, and the 10–90% interval as whiskers.

### Reporting summary

Further information on research design is available in the Nature Portfolio Reporting Summary linked to this article.

## Supplementary information

Supplementary Information

Description of Additional Supplementary Files

Supplementary Data 1

Supplementary Data 2

Supplementary Data 3

Supplementary Data 4

Supplementary Data 5

Supplementary Data 6

Reporting Summary

## Data Availability

The used plasmids and raw data can be shared upon reasonable requests.

## Code availability

All used fitting procedures were standard and are mentioned in the text.
