# Supplementary material for: Kinetic fingerprinting of metabotropic glutamate receptors
Source: Commun Biol. 2023 Jan 27;6:104. doi: 10.1038/s42003-023-04468-z (PMC9883448; doi:10.1038/s42003-023-04468-z)
Supplement: Supplementary file 1 — Supplementary Information [file 42003_2023_4468_MOESM1_ESM.pdf]

Supplementary Information

to the manuscript

Kinetic fingerprinting of metabotropic glutamate receptors

by Taulant Kukaj, Christian Sattler, Thomas Zimmer, Ralf Schmauder, Klaus Benndorf

Institute of Physiology II, Jena University Hospital, Friedrich Schiller University Jena, 07743 Jena, Germany

Includes

Supplementary Discussion

Supplementary Figure 1 to 15

Supplementary Tables 1 to 4

Supplementary Discussion

When considering the sequence alignment of the eight mGluRs (Supplementary Fig. 1), we noticed a peculiarity which seems to correspond to our experimental results: In LBD1a, comprising the dimerization-relevant helices B and C[4], there are 27.4% identical positions in all mGluRs (left gray column in Supplementary Fig. 13), which cannot be responsible for the observed functional differences. However, when considering identical positions in the mGluRs of two groups when at least one position in the third group differs, there is a pronounced difference between the three cases. When positions in group I and II are conserved while at least one position in group III differs, this fraction was 6.94% (left green column in Supplementary Fig. 13) and when positions in group II and III are conserved while at least one position in group I differs, this fraction was 13.29% (left red column in Supplementary Fig. 13). In contrast, when positions in group I and III are conserved while at least one position in group III differs, we identified only one position, resulting in a fraction of 0.58% (left blue column in Supplementary Fig. 13). Hence, the elevated propensity found in our kinetic results for heterodimers of group I and II and group II and III is to some extent mirrored by this comparison of the sequences. In this logic a remarkable observation is also that in LBD2b only the case with conserved positions in group II and III appears when at least one position in group I differs (Supplementary Fig. 13). It remains to be left for future structural analyses to substantiate analyze whether or not some of these peculiarities in the sequence comparison cause indeed the differences in the propensity of the different subunits.

Supplementary Figures

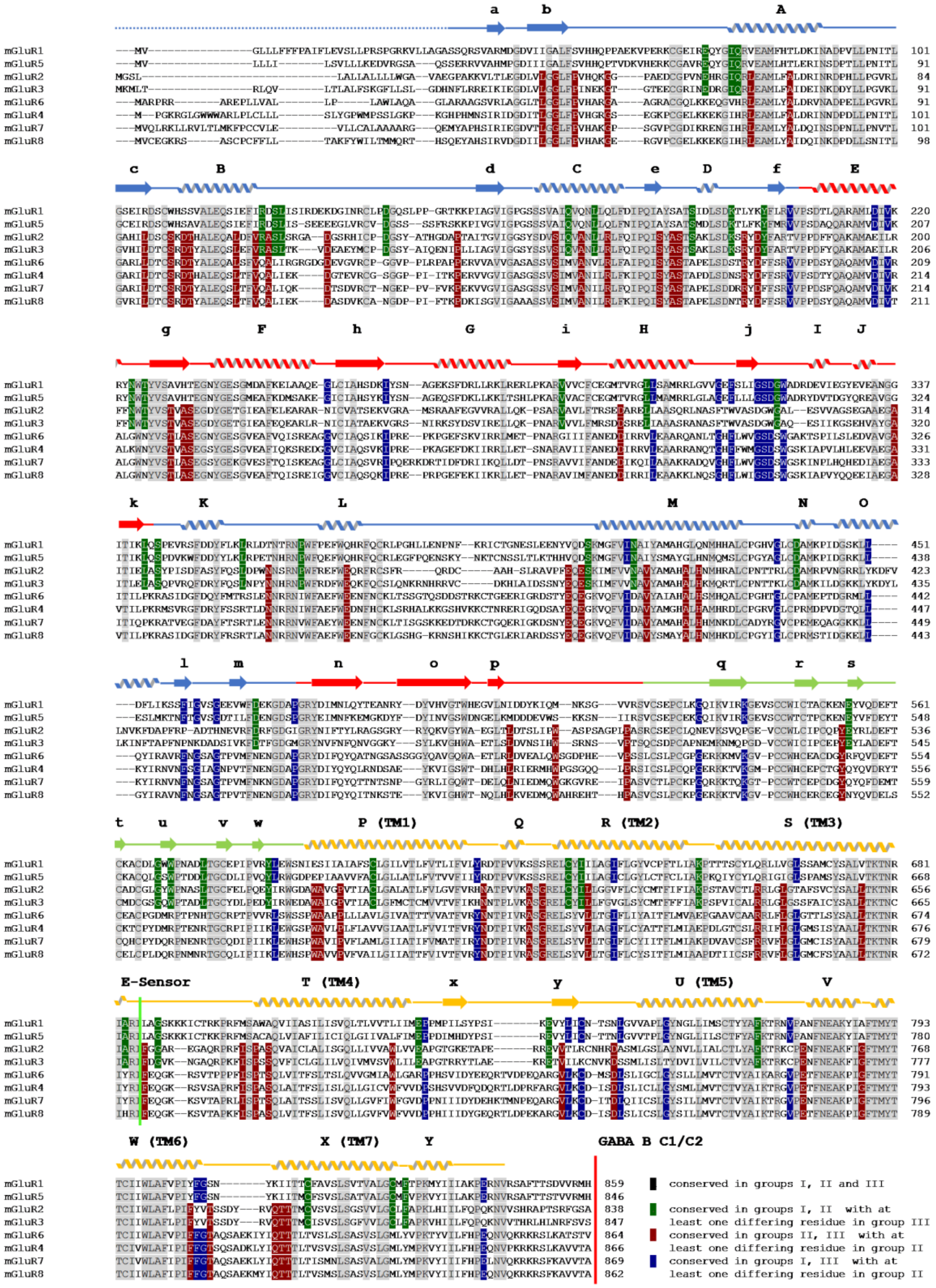

Supplementary Fig. 1 Full-length human mGluR1 to mGluR8 in the sequence given by the phylogenetic tree (Fig. 5a). Secondary structures are represented by helices (α helices A-Y), arrows (β strands a-y), straight lines (loops, combining elements between secondary structures etc.), dotted lines (no information available). Symbols: blue - ligand binding domain 1, red - ligand binding domain 2, green - cysteine rich domain, orange - transmembrane domains, yellow box - amino acids involved in binding, bright green vertical line - position of the E-sensor, red vertical line - cutting sites for the constructs. Conserved residues are labeled according to the indicated color code. The following entries were used: mGluR1, Q14832; mGluR2, Q14416; mGluR3, Q14832; mGluR4, Q14833; mGluR5, P41594; mGluR6, Q15303; mGluR7, Q14831; mGluR8, O00222. The C-termini not included in our constructs are omitted from the alignment, constructs without the $GABA_B$ sequence have truncated C-terminal tails as well.

mGluR	1	5	2	3	6	4	7	8
1	100							
5	61.8	100						
2	43.9	43.6	100					
3	42.6	42.5	68.6	100				
6	40.5	39.6	46.4	45.9	100			
4	41.4	41.0	46.1	45.7	70.1	100		
7	39.7	39.4	44.3	43.9	67.1	69.6	100	
8	40.7	41.2	44.6	44.9	70.4	75.0	73.8	100

Supplementary Fig. 2 Sequence identity among the eight mGluRs. The numbers are given in percent.

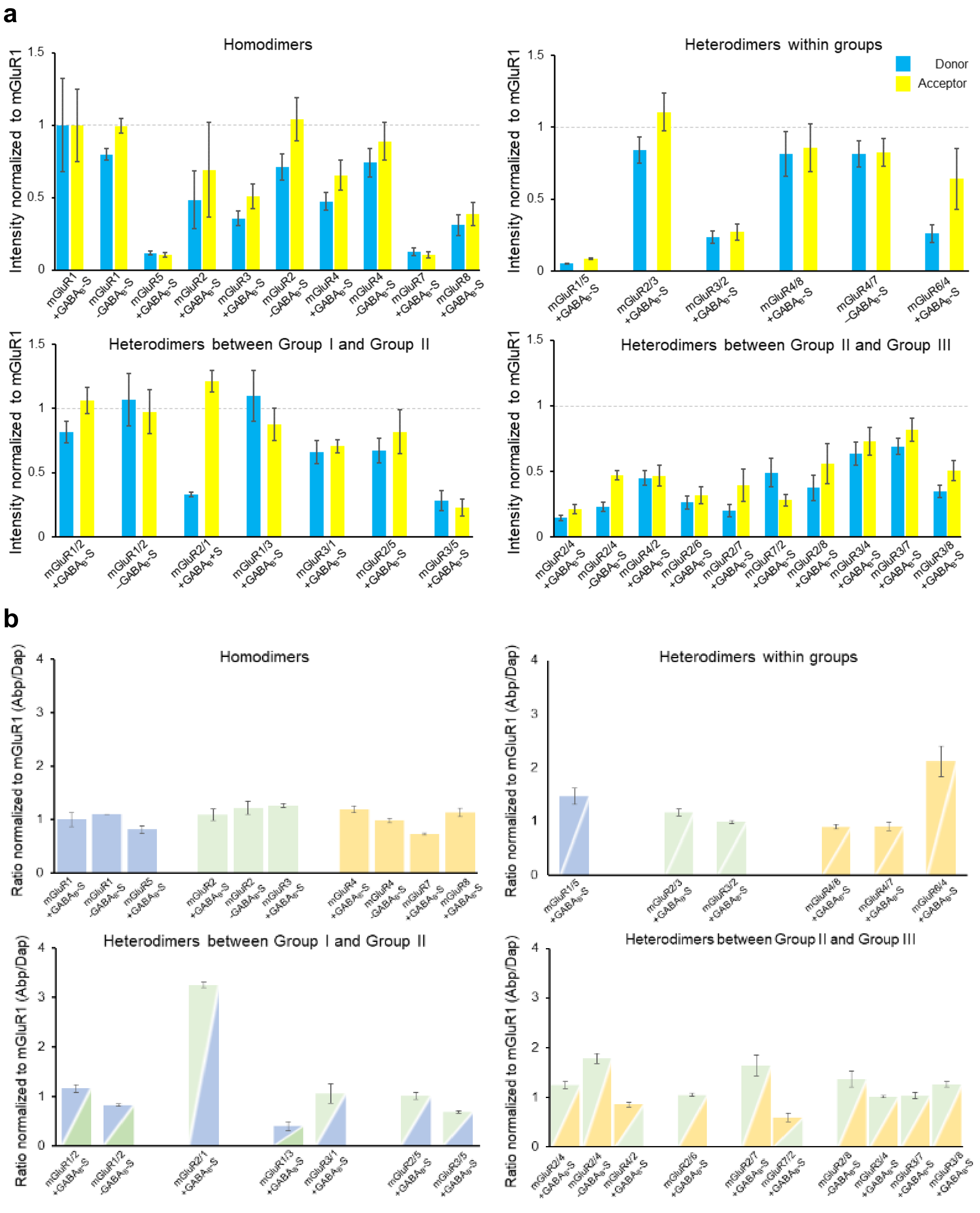

Supplementary Fig. 3 Relative expression of mGluRs. ROIs of photobleaching experiments on oocyte membranes were evaluated. **a** The donor (cyan) signal was considered after photobleaching. The acceptor-signal (yellow) was evaluated before photobleaching. All data are corrected for measurement conditions (e.g. laser power) and normalized to mGluR1 expression. **b** Relative ratios of the expression levels (acceptor/donor) normalized to mGluR1. A failure to retain the GABA-C1 (donor) within the ER leads to reduced ratios. A failure of the GABA-C2 (acceptor) to mask GABA-C1 and release it from the ER leads to elevated ratios.

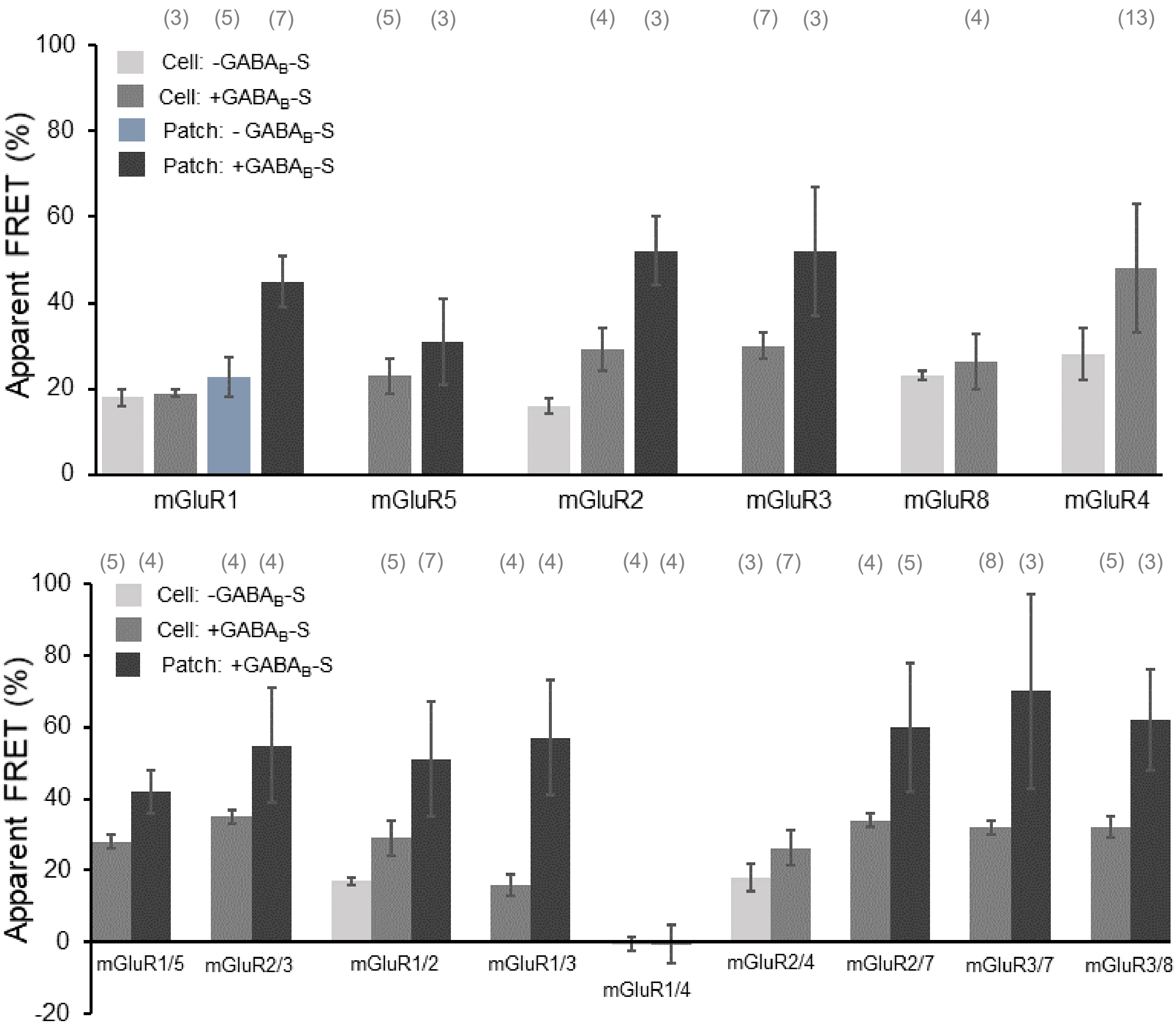

Supplementary Fig. 4 Apparent basal FRET efficiencies: Basal FRET-efficiencies were estimated in cells from donor-dequenching upon acceptor-photobleaching experiments or in patches from time-series with concentrations-jumps of glutamate (see Supplementary Methods). Whole-cell experiments had only poor optical resolution to exclude any intracellular signal, or auto-fluorescence, and are thus a lower estimate. The GABA-system enriches donor:acceptor dimers and thus increases apparent FRET-efficiencies both in whole cells and isolated membrane patches. Patches without GABA therefore might show systematic errors due to non-FRET donor:donor dimer background.

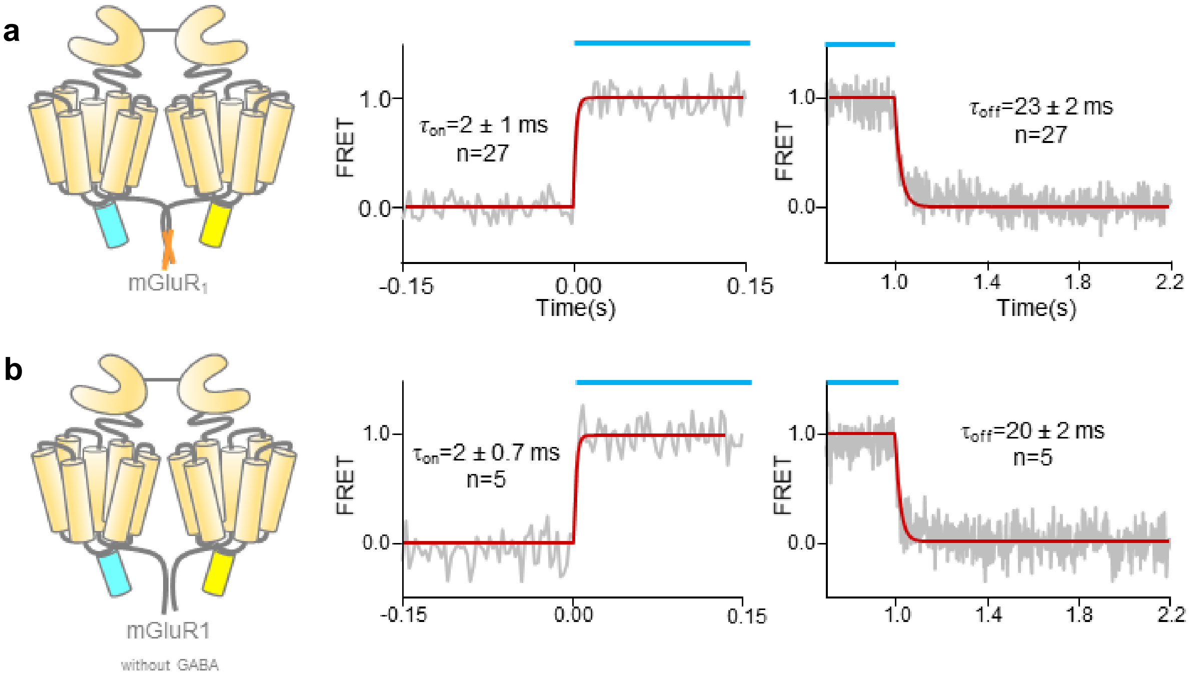

Supplementary Fig. 5 Kinetics in dimeric mGluR1 in the presence and absence of GABA$_B$ tails. **a**, **b** Lack of effect of the GABA$_B$ tails on signal kinetics. For the example of mGluR1/1, generating the largest signals, activation and deactivation were similar with (b) and without the GABA$_B$ tails (c). The light blue bars indicate exposure to 1 mM glutamate.

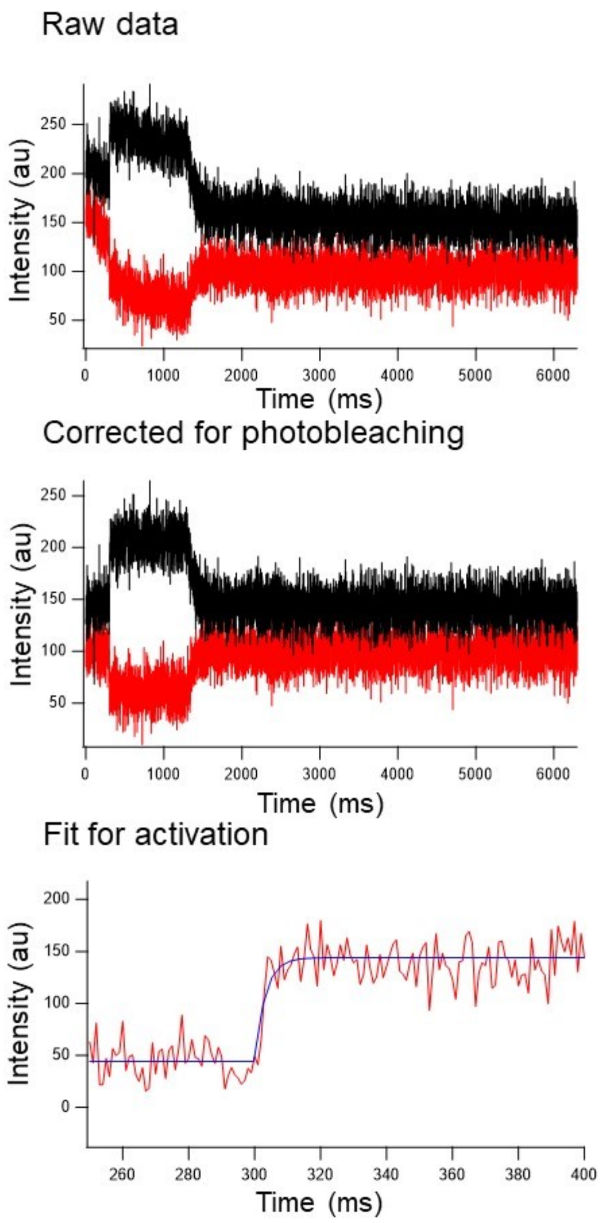

Supplementary Fig. 6. Calculation of FRET. Raw data showing photo-bleaching. The time interval containing the concentration jump and 2.5 seconds accommodating the deactivation kinetics was masked and the remaining trace was fitted with mono-(for the donor) or bi-exponential functions (FRET signal in the acceptor channel). One component of the FRET/acceptor bleaching was set to the donor-bleaching time constant. The time constants remained fixed within one measurement. Activation and deactivation kinetics in the calculated FRET traces were then fitted separately. Time ranges of the fits were from jump - 100 ms to jump + 300 ms and from jump -100 ms to jump +1000 ms for activation and deactivation, respectively. For the deactivation of mGluR1 fits were repeated with the time range jump - 100 ms to jump + 300 ms to reduce the influence of baseline noise and drift.

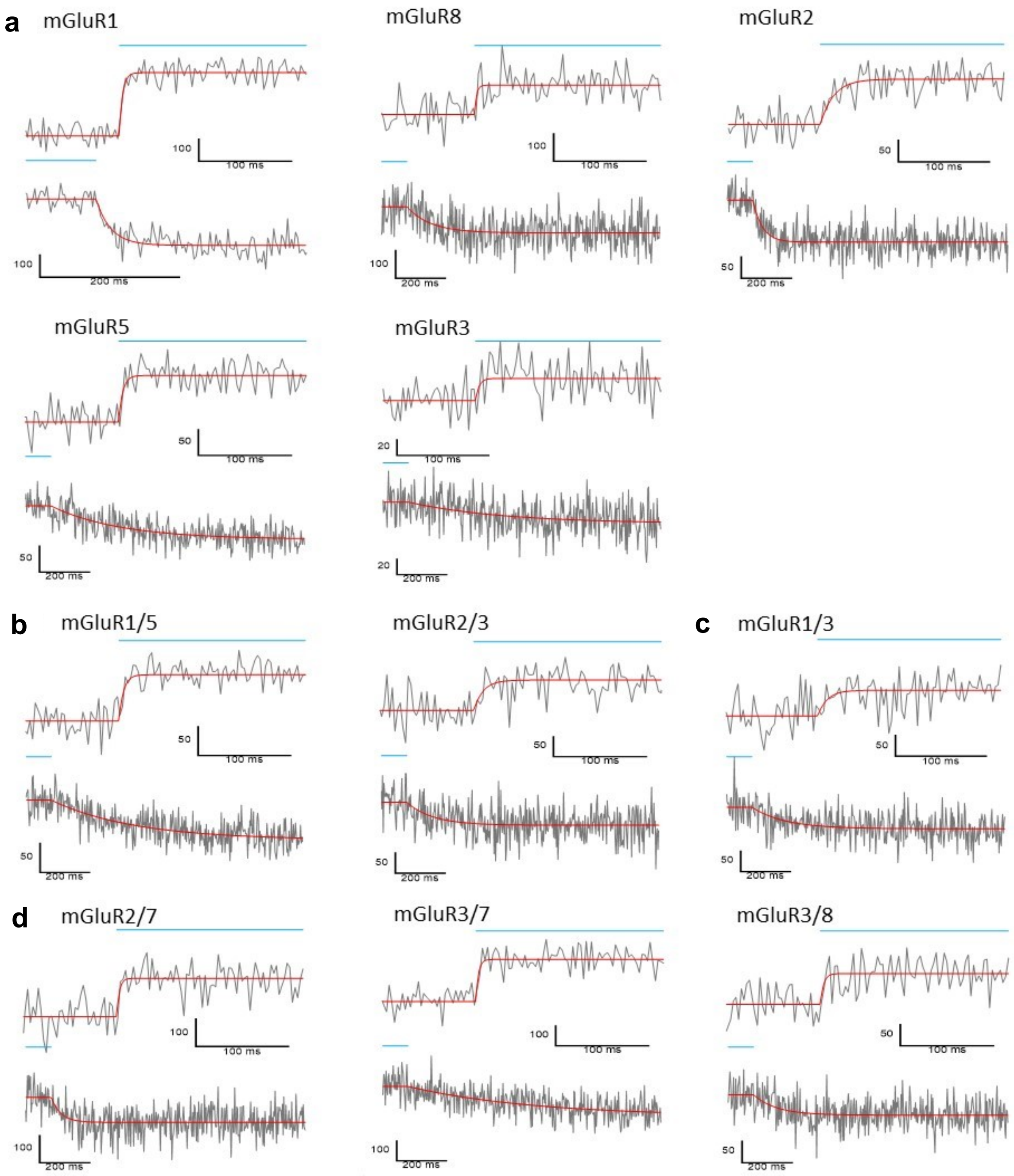

	τ_{on}	SEM	τ_{off}	SEM
mGluR1	3.4	0.6	21	5
mGluR5	4.2	1.8	273	39
mGluR1/5	4.8	1.8	332	50
mGluR2	14.4	3.6	38	7
mGluR3	3.7	4.9	378	153
mGluR2/3	9.9	5.1	104	28
mGluR1/3	10.3	6.9	132	31
mGluR8	2.0	2.1	95	23
mGluR2/7	2.8	2.1	38	12
mGluR3/7	2.6	1.3	502	140
mGluR3/8	3.3	3.0	91	26

Supplementary Fig. 7. Examples of activation and deactivation kinetics for the constructs analyzed kinetically. **a** Five homodimers. **b** Heterodimers between mGluRs within a group, mGluR1/5 and mGluR2/3. **c** Heterodimers between group I and II: mGluR1/3. **d** Heterodimers between group II and III, mGluR2/7, mGluR3/7, and mGluR3/8. The table gives fit-results and errors for the shown examples. Overall statistics are shown in Table 4. The light blue bars indicate glutamate application of the concentrations provided by Supplementary Table 1.

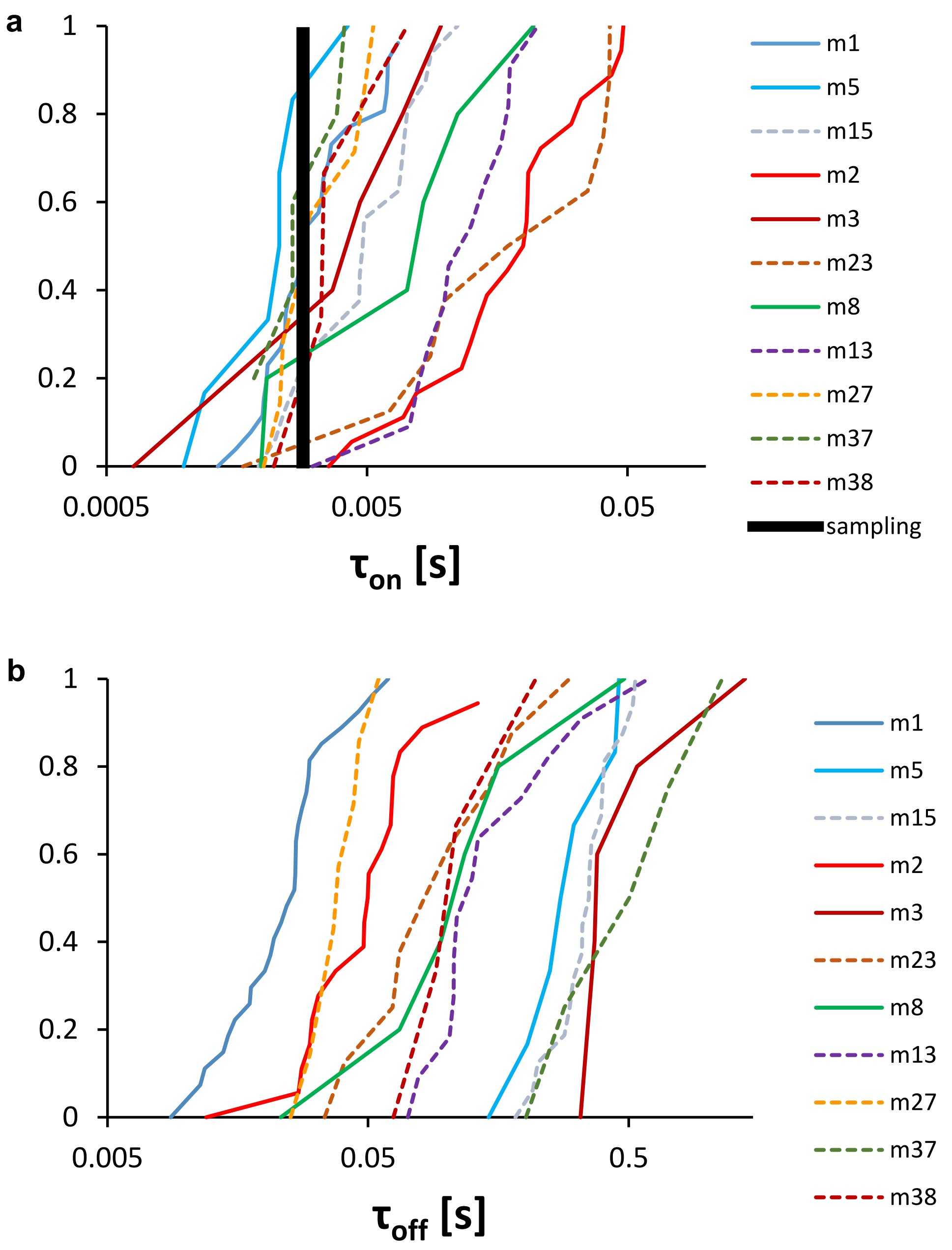

Supplementary Fig. 8. Fit results of all traces passing the selection criteria. a Cumulative distribution off all fit results upon applying glutamate (activation). Black dashed lines indicate the sampling interval of the measurements, values below or near this value are purely resolved. **b** Cumulative distribution off all fit results upon glutamate removal (deactivation). Sampling Interval is short/outside the plotted ordinate. Dashed lines indicate heterodimers, solid lines homodimers.

	1	5	2	3	4	6	7	8
1	19 ± 2							
5	29 ± 5	23 ± 4						
2	29 ± 5	18 ± 3	26 ± 4					
3	16 ± 3	21 ± 3	35 ± 2	30 ± 3				
4	-	-	35 ± 5	20 ± 1	18 ± 1			
6	-	-	26 ± 3	-	-	-		
7	21 ± 3	19 ± 4	34 ± 2	32 ± 2	-	-	18 ± 5	
8	-	-	35 ± 1	32 ± 3	13 ± 1	-	-	28 ± 6

Kinetics with evaluable time courses

Kinetics with non-evaluable time courses

Supplementary Fig. 9 FRET efficiency in heterodimeric mGluRs. FRET efficiency by donor dequenching (in % ± SEM) is indicated in a matrix. The values were obtained from 3 to 10 cells. The main diagonal represents the values of the homodimers. For better comparison, combinations with both evaluable and non-evaluable kinetics are indicated by colors. White fields containing numbers indicate heterodimers with no observable ligand responses. Empty fields indicate that no donor dequenching was observed upon acceptor photo-bleaching.

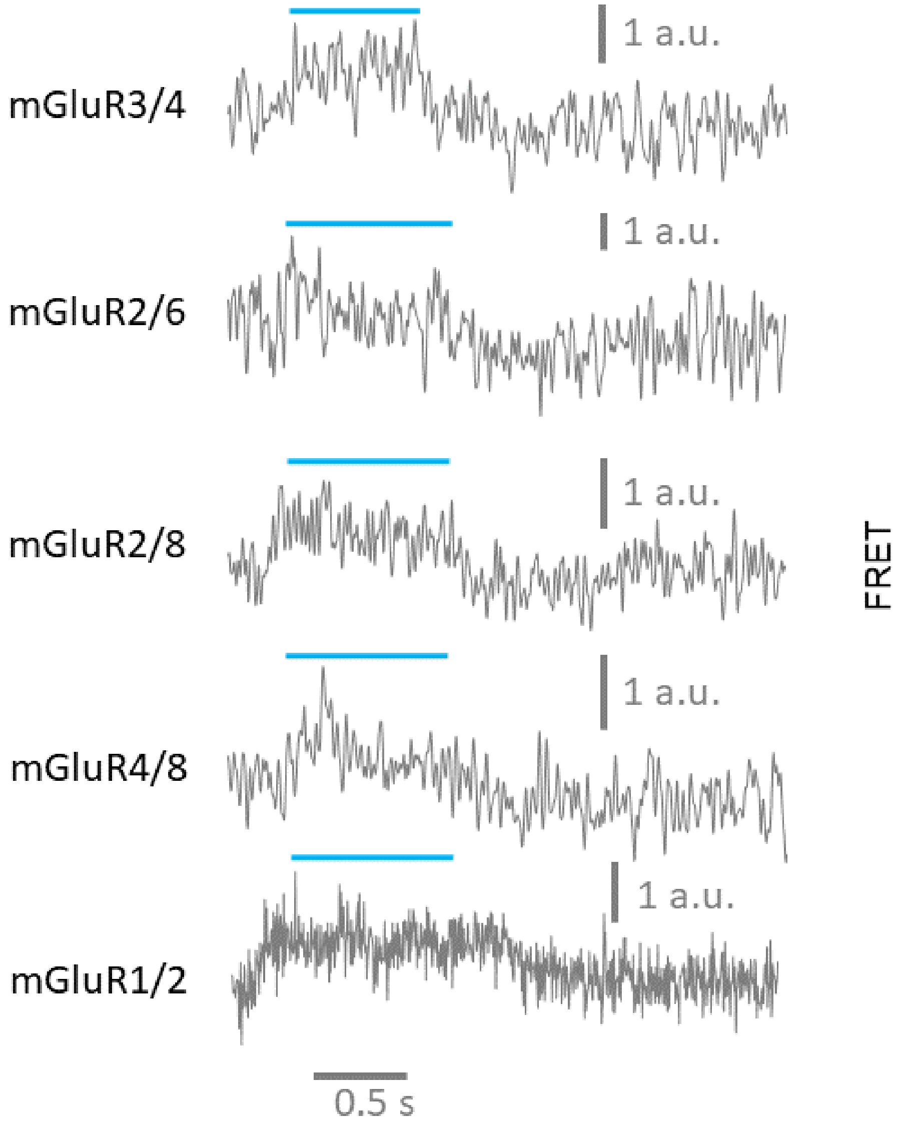

Supplementary Fig. 10: Heterodimers showing FRET changes too small for quantifying time courses. The five heterodimers mGluR3/4, mGluR2/6, mGluR2/8, mGlu4/8 and mGluR1/2 provided time-dependent responses too small to be subjected to kinetic analysis, proving, nevertheless, functionality of the heterodimers.

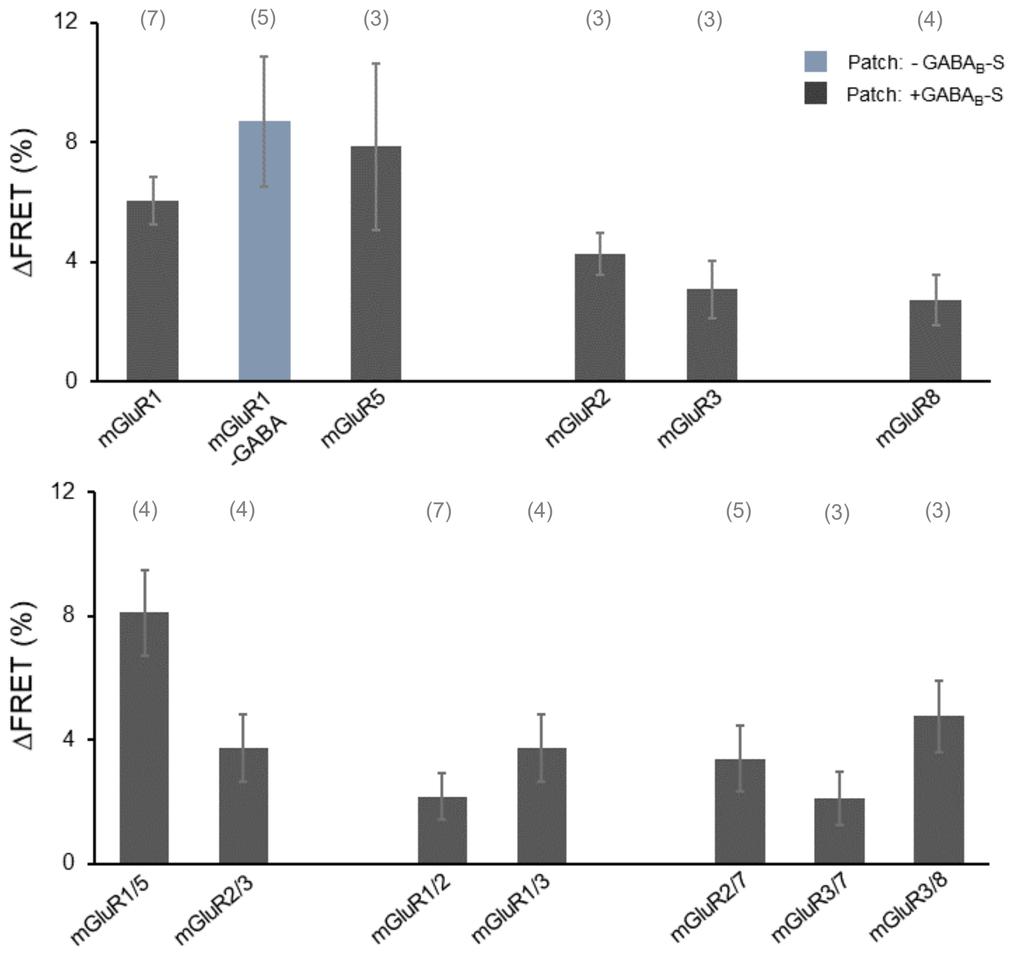

Supplementary Fig. 11 FRET changes upon glutamate binding. FRET-efficiencies change as from time-series with concentration-jumps of glutamate (see Supplementary Methods). This method assumes a 1:1 stoichiometry of donor and FRET active acceptor, thus the '-GABA' values are a lower estimate.

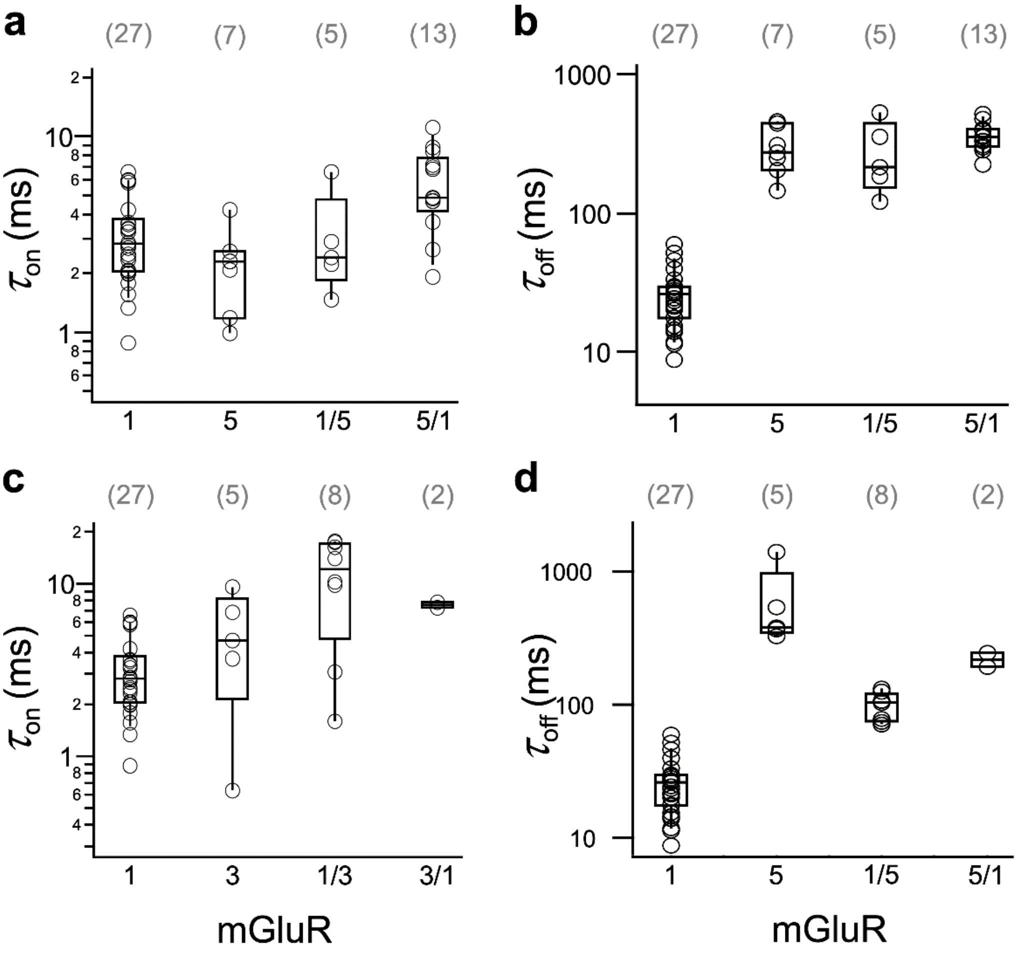

Supplementary Fig. 12 Comparison of the kinetics at exchanged fluorescence label and $GABA_B$ sequence. To exclude functional effects of the chosen combination of fluorescence label (CFP or YFP) and the $GABA_B$ sequence of the quality control system (C1 and C2) (see Methods), two heterodimers were tested as examples when swapping both sequences among the subunits. The result was that neither activation nor deactivation kinetics were different. **a** mGluR1-CFP-C1/mGluR5-YFP-C2 versus mGluR5-YFP-C2/mGluR5-CFP-C1. **b** mGluR1-CFP-C1/mGluR3-YFP-C2 versus mGluR3-CFP-C1/mGluR1-YFP-C2.

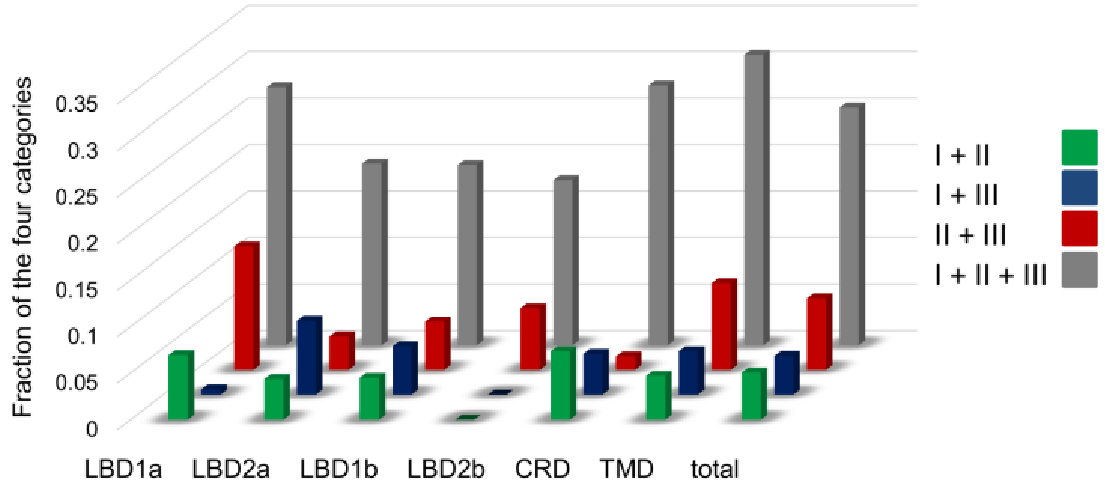

Supplementary Fig. 13 Comparison of identical amino acids in groups I, II and III. The frequency of different identities as fraction of unity are plotted for the two parts of the ligand binding domain 1 (LBD1 a, b), of the ligand binding domain 2 (LBD2 a, b), the cysteine-rich domain (CRD) and the transmembrane domain (TMD). Gray: Conserved in all three groups; green: conserved in group I and II with at least one differing amino acid in group III; blue: conserved in group I and III with at least one differing amino acid in group II; red: conserved in group II and III with at least one differing amino acid in group I.

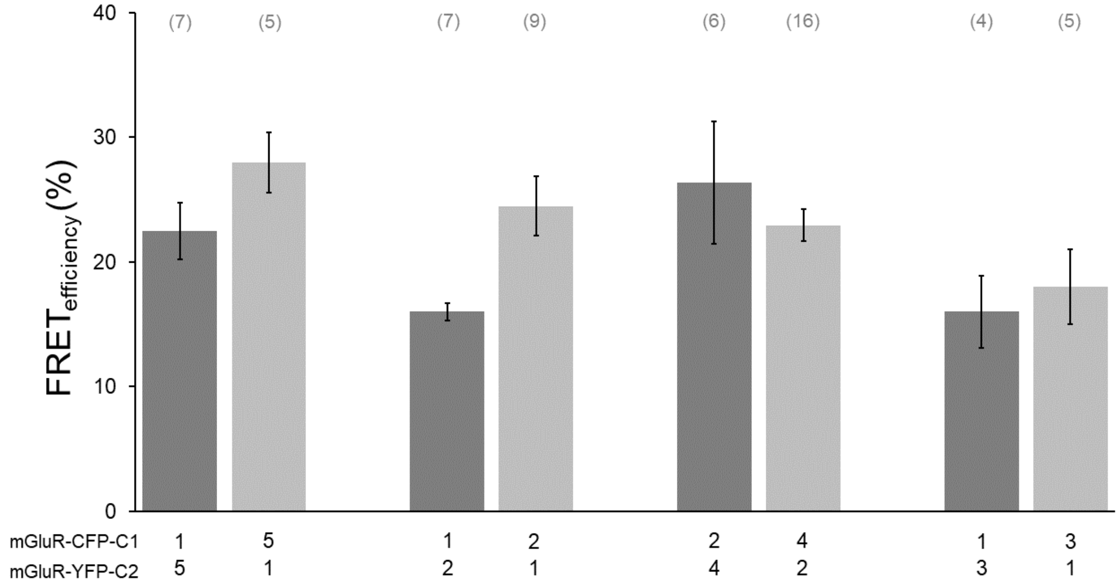

Supplementary Fig. 14 Comparison of the FRET efficiency at exchanged fluorescence label and $GABA_B$ sequence: To demonstrate whether exchanged fluorophores and $GABA_B$ sequence might play a role in FRET efficiency, we tested dimerization by measuring FRET efficiency after acceptor photobleaching for some selected dimers. We selected four examples, mGluR1/mGluR5, mGluR1/mGluR3, mGluR1/mGluR2, and mGluR2/mGluR4. FRET efficiency was not significantly different as expected for mGluR1/5, mGluR1/3 and mGluR2/4 combinations. An exception was observed for mGluR1/2, where mGluR-CFP-C1 in combination with mGluR2-YFP-C2 showed lower FRET values compared to mGluR2-CFP-C1 and mGluR1-YFP-C2. This suggests that mGluR1 expresses better than mGluR2, therefore some of the mGluR1-CFP-C1 escape the ER retention system and express as the mGluR1-CFP-C1 homodimer, thereby increasing the background noise. The numbers of experiments are shown at the top in brackets.

a

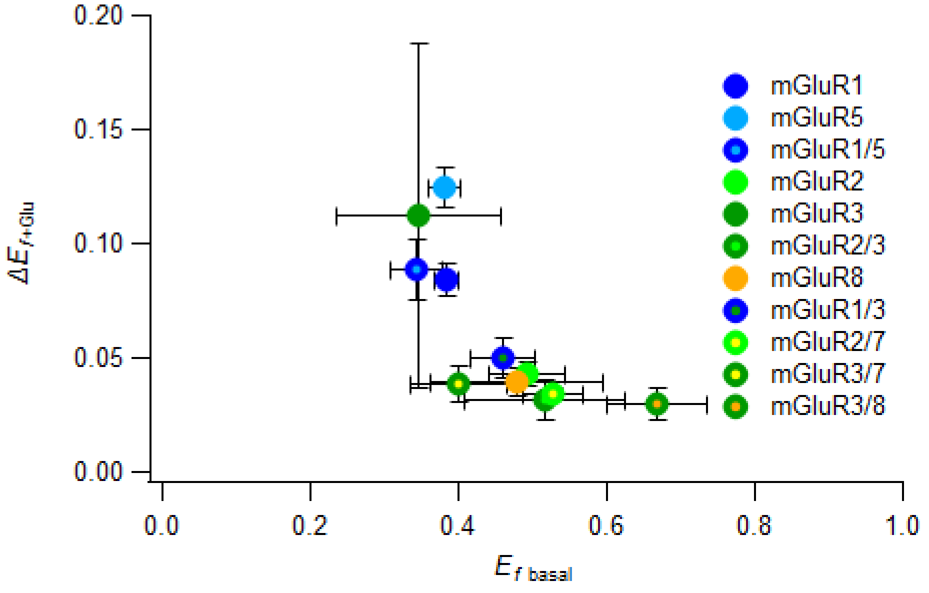

b

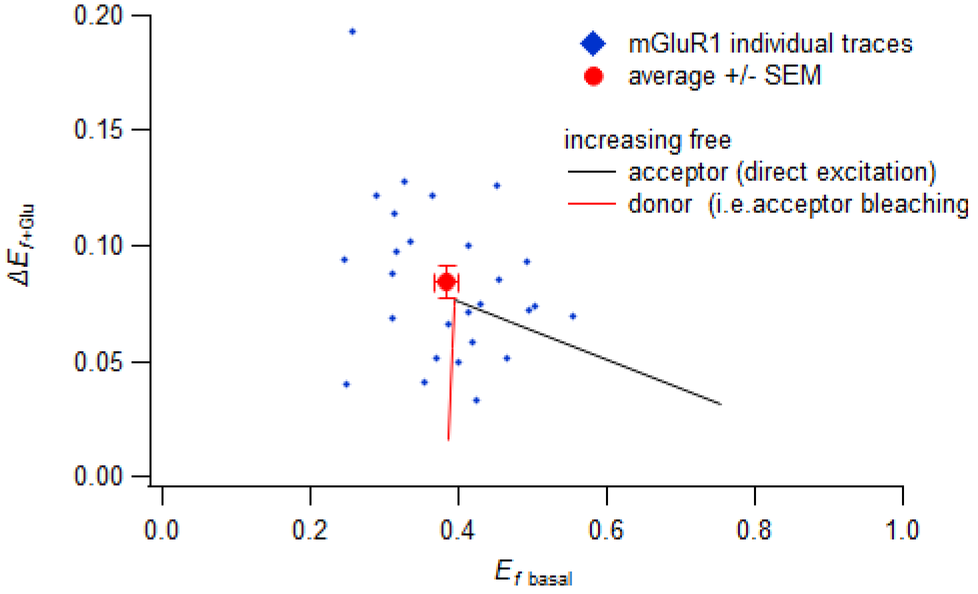

Supplementary Fig 15. Basal FRET and FRET changes. a FRET-changes vs. basal FRET for all analyzed subtypes. Only the first repetition of each experiment was evaluated to reduce artifacts due to bleached fluorophores. **b** Values for individual experiments on the example of mGluR1. Effect of additional free acceptor signal in the FRET channel, e.g. due to direct excitation (black line), and the effect of additional free donor signal, e.g. due to bleached acceptor, was simulated. No correlation between FRET-changes and basal FRET consistent with such artifacts was observed.

Supplementary Tables:

	C_{used} (µM)	Maximum EC_{50} (µM) (published)	Ratio: C_{used}/EC_{50} (published)
**mGluR1**	1,000	13	77
**mGluR5**	1,000	11	91
**mGluR2**	1,000	12	83
**mGluR3**	1,000	9	111
**mGluR7**	10,000	2300	4
**mGluR8**	1,000	10	100
**mGluR1/5**	1,000	20	50
**mGluR2/3**	1,000	Between mGluR2 and 3	
**mGluR2/7**	1,000	2,7	370
**mGluR3/7**	1,000		
**mGluR3/8**	1,000		

Supplementary Table 1 Comparison of concentrations used in this study and published EC_{50} values. Published EC_{50}-Values: Sources: Homodimers[2]. Heterodimers: mGluR2/7[3], mGluR2/3[4,5]. Care was taken to measure kinetics at highly saturating concentrations, where the contribution of binding kinetics on the activation rate was assumed to be negligible. An exception was mGluR7, as concentrations in excess to 10 mM interfere with patch stability.

t_{on}	group	N	log(t_{on}[s])	SEM	t_{on}[ms]	p-Values		Turkey-Kramer								
**mGluR1**	I	27	-2.54	0.05	2.9	x	9.8E-01	3.4E-01	5.9E-13	1.0E+00	9.8E-08	2.5E-01	2.2E-06	1.0E+00	1.0E+00	1.0E+00
**mGluR5**	I	7	-2.7	0.1	2.0	9.8E-01	x	1.5E-01	3.6E-09	8.8E-01	1.1E-06	9.2E-02	2.0E-05	9.6E-01	1.0E+00	9.4E-01
**mGluR1/5**	I/I	17	-2.33	0.07	4.7	3.4E-01	1.5E-01	x	4.3E-06	1.0E+00	1.9E-03	1.0E+00	3.4E-02	9.5E-01	9.2E-01	1.0E+00
**mGluR2**	II	19	-1.78	0.07	16.7	5.9E-13	3.6E-09	4.3E-06	x	7.6E-04	1.0E+00	6.3E-02	8.2E-01	1.7E-06	2.9E-05	2.6E-03
**mGluR3**	II	5	-2.4	0.1	3.7	1.0E+00	8.8E-01	1.0E+00	7.6E-04	x	1.0E-02	9.7E-01	8.6E-02	1.0E+00	1.0E+00	1.0E+00
**mGluR2/3**	II/II	9	-1.8	0.1	15.0	9.8E-08	1.1E-06	1.9E-03	1.0E+00	1.0E-02	x	2.9E-01	9.9E-01	2.1E-04	7.7E-04	2.1E-02
**mGluR8**	III	6	-2.2	0.1	6.2	2.5E-01	9.2E-02	1.0E+00	6.3E-02	9.7E-01	2.9E-01	x	8.1E-01	7.3E-01	6.9E-01	9.7E-01
**mGluR1/3**	I/II	12	-1.96	0.08	11.0	2.2E-06	2.0E-05	3.4E-02	8.2E-01	8.6E-02	9.9E-01	8.1E-01	x	3.4E-03	9.1E-03	1.4E-01
**mGluR2/7**	II/III	8	-2.5	0.1	3.2	1.0E+00	9.6E-01	9.5E-01	1.7E-06	1.0E+00	2.1E-04	7.3E-01	3.4E-03	x	1.0E+00	1.0E+00
**mGluR3/7**	II/III	5	-2.5	0.1	2.9	1.0E+00	1.0E+00	9.2E-01	2.9E-05	1.0E+00	7.7E-04	6.9E-01	9.1E-03	1.0E+00	x	1.0E+00
**mGluR3/8**	II/III	4	-2.4	0.1	3.7	1.0E+00	9.4E-01	1.0E+00	2.6E-03	1.0E+00	2.1E-02	9.7E-01	1.4E-01	1.0E+00	1.0E+00	x
						**mGluR1**	**mGluR5**	**mGluR1/5**	**mGluR2**	**mGluR3**	**mGluR2/3**	**mGluR8**	**mGluR1/3**	**mGluR2/7**	**mGluR3/7**	**mGluR3/8**

Supplementary Table 2. Fit results for τ_{on} , number of experiments and statistical relevant differences between log(τ_{on}) for homo- and hetero-dimeric mGluRs: For plots and statistical test log(τ_{on}) for the number of values N were evaluated, one-factor ANOVA was followed by Turkey-Kramer post-hoc test, blue indicates significant difference (light blue $p<0.05$; dark blue $p<0.01$). Value above and below the diagonal are identical.

t_{off}	group	N	$log(t_{off}[s])$	SEM	$t_{off}[ms]$	p-Values mGluR1	mGluR5	Turkey-Kramer mGluR1/5	mGluR2	mGluR3	mGluR2/3	mGluR8	mGluR1/3	mGluR2/7	mGluR3/7	mGluR3/8
mGluR1	I	27	-1.63	0.05	23	x	2.2E-14	2.2E-14	1.3E-03	2.2E-14	2.6E-07	1.1E-06	3.4E-14	5.5E-01	2.2E-14	4.5E-05
mGluR5	I	7	-0.56	0.09	277	2.2E-14	x	1.0E+00	3.4E-09	7.1E-01	3.5E-03	6.0E-02	3.6E-01	8.2E-09	8.5E-01	1.9E-01
mGluR1/5	I/I	17	-0.47	0.06	337	2.2E-14	1.0E+00	x	9.9E-01	0.0E+00	9.9E-01	0.0E+00	9.5E-01	0.0E+00	0.0E+00	0.0E+00
mGluR2	II	19	-1.32	0.05	48	1.3E-03	3.4E-09	9.9E-01	x	3.6E-12	1.7E-01	9.9E-02	9.6E-06	9.9E-01	1.6E-11	2.1E-01
mGluR3	II	5	-0.3	0.1	510	2.2E-14	7.1E-01	0.0E+00	3.6E-12	x	5.1E-06	2.6E-04	2.0E-03	1.1E-11	1.0E+00	2.3E-03
mGluR2/3	II/II	9	-1.05	0.08	89	2.6E-07	3.5E-03	9.9E-01	1.7E-01	5.1E-06	x	1.0E+00	5.9E-01	5.2E-02	1.6E-05	1.0E+00
mGluR8	III	6	-1.0	0.1	104	1.1E-06	6.0E-02	0.0E+00	9.9E-02	2.6E-04	1.0E+00	x	9.7E-01	2.9E-02	6.7E-04	1.0E+00
mGluR1/3	I/II	12	-0.83	0.07	148	3.4E-14	3.6E-01	9.5E-01	9.6E-06	2.0E-03	5.9E-01	9.7E-01	x	1.4E-05	5.4E-03	1.0E+00
mGluR2/7	II/III	8	-1.43	0.08	38	5.5E-01	8.2E-09	0.0E+00	9.9E-01	1.1E-11	5.2E-02	2.9E-02	1.4E-05	x	4.2E-11	7.0E-02
mGluR3/7	II/III	5	-0.3	0.1	471	2.2E-14	8.5E-01	0.0E+00	1.6E-11	1.0E+00	1.6E-05	6.7E-04	5.4E-03	4.2E-11	x	4.9E-03
mGluR3/8	II/III	4	-1.0	0.1	108	4.5E-05	1.9E-01	0.0E+00	2.1E-01	2.3E-03	1.0E+00	1.0E+00	1.0E+00	7.0E-02	4.9E-03	x

Supplementary Table 3: Fit results for τ_{off} , number ox experiments and statistical relevant differences between $\log(\tau_{off})$ for homo- and hetero-dimeric mGluRs: For plots and statistical test $\log(\tau_{off})$ for the number of values N were evaluated, one-factor ANOVA was followed by Turkey-Kramer post-hoc test, blue indicates significant difference (light blue $p<0.05$; dark blue $p<0.01$). Value above and below the diagonal are identical.

	group	N in analysis	N performed	fraction
mGluR1	I	27	58	0.47
mGluR5	I	7	13	0.54
mGluR1/5	I/I	17	44	0.39
mGluR2	II	19	74	0.26
mGluR3	II	5	128	0.04
mGluR2/3	II/II	9	61	0.15
mGluR8	III	6	65	0.09
mGluR1/3	I/II	12	31	0.39
mGluR2/7	II/III	8	33	0.24
mGluR3/7	II/III	5	26	0.19
mGluR3/8	II/III	4	15	0.27
mGluR4	III		15	
mGluR7	III		16	
mGluR1/2	I/II	1	30	0.03
mGluR1/6	I/III		2	
mGluR1/7	I/III		13	
mGluR2/4	II/III		11	
mGluR2/5	II/III		11	
mGluR2/6	II/III		7	
mGluR2/8	II/III	2	25	0.08
mGluR3/4	II/III		15	
mGluR4/7	III/III		4	
mGluR4/8	III/III		15	
mGluR7/8	III/III		6	
total		122	718	0.17

Supplementary Table 4: Number of experiments performed and included in the evaluation.

Supplementary References

1. Leys C, Ley C, Klein O, Bernard P, Licata L. Detecting outliers: Do not use standard deviation around the mean, use absolute deviation around the median. *J Exp Soc Psychol* **49**, 764-766 (2013)

2. Acher, F.C., Metabotropic glutamate receptors. *Tocris Biosci Rev Lett* **26**, 1–10 (2011).

3. Habrian CH*, et al.* Conformational pathway provides unique sensitivity to a synaptic mGluR. *Nature communications* **10**, 5572 (2019).

4. Levitz J, Habrian C, Bharill S, Fu Z, Vafabakhsh R, Isacoff EY. Mechanism of Assembly and Cooperativity of Homomeric and Heteromeric Metabotropic Glutamate Receptors. *Neuron* **92**, 143-159 (2016).

5. Lee J*, et al.* Defining the Homo- and Heterodimerization Propensities of Metabotropic Glutamate Receptors. *Cell Rep* **31**, 107605 (2020).
