# Supplementary material for: Kinetic fingerprinting of metabotropic glutamate receptors
Source: Commun Biol. 2023 Jan 27;6:104. doi: 10.1038/s42003-023-04468-z (PMC9883448; doi:10.1038/s42003-023-04468-z)
Supplement: Supplementary file 2 — Description of Additional Supplementary Files [file 42003_2023_4468_MOESM2_ESM.pdf]

Description of Additional Supplementary Files

File name: Supplementary Data 1

Description: Source data for Figure 1

File name: Supplementary Data 2

Description: Source data for Figure 2

File name: Supplementary Data 3

Description: Source data for Figure 3

File name: Supplementary Data 4

Description: Source data for Figure 4

File name: Supplementary Data 5

Description: Source data for Figure 5

File name: Supplementary Data 6

Description: Source data for Supplementary Figures
